# FRET Sensor-Modified Synthetic Hydrogels for Real-Time Monitoring of Cell-Derived Matrix Metalloproteinase Activity using Fluorescence Lifetime Imaging

**DOI:** 10.1002/adfm.202309711

**Published:** 2024-01-30

**Authors:** Ziqian Yan, Thomas Kavanagh, Ricardo da Silva Harrabi, Suzette T. Lust, Chunling Tang, Rebecca Beavil, Manuel M. Müller, Andrew Beavil, Simon Ameer-Beg, Ricardo M.P. da Silva, Eileen Gentleman

**Affiliations:** 1 Centre for Craniofacial and Regenerative Biology, King’s College London, London, UK; 2 Richard Dimbleby Laboratory of Cancer Research, School of Cancer and Pharmaceutical Sciences, King’s College London, London, UK; 3 Randall Centre of Cell and Molecular Biophysics, King’s College London, London, UK; 4 Department of Chemistry, King’s College London, London, UK; 5 Department of Biomedical Sciences, University of Lausanne, Lausanne, Switzerland

**Keywords:** Matrix metalloproteinase, hydrogel, matrix remodeling, FRET sensor

## Abstract

Matrix remodeling plays central roles in a range of physiological and pathological processes and is driven predominantly by the activity of matrix metalloproteinases (MMPs), which degrade extracellular matrix (ECM) proteins. Our understanding of how MMPs regulate cell and tissue dynamics is often incomplete as *in vivo* approaches are lacking and many *in vitro* strategies cannot provide high-resolution, quantitative measures of enzyme activity *in situ* within tissue-like 3D microenvironments. Here, we incorporate a Förster resonance energy transfer (FRET) sensor of MMP activity into fully synthetic hydrogels that mimic many properties of the native ECM. We then use fluorescence lifetime imaging to provide a real-time, fluorophore concentration-independent quantification of MMP activity, establishing a highly accurate, readily adaptable platform for studying MMP dynamics *in situ*. MCF7 human breast cancer cells encapsulated within hydrogels highlight the detection of MMP activity both locally, at the sub-micron level, and within the bulk hydrogel. Our versatile platform may find use in a range of biological studies to explore questions in the dynamics of cancer metastasis, development, and tissue repair by providing high-resolution, quantitative and *in situ* readouts of local MMP activity within native tissue-like environments.

## 1 Introduction

Matrix remodeling, the process by which cells actively degrade and form new extracellular matrix (ECM), plays fundamental roles in tissue growth, homeostasis, and repair. However, when matrix remodeling goes awry, it can contribute to numerous pathologies, most notably fibrosis, which plays a role in up to 45% of deaths worldwide. ^[1]^ Matrix metalloproteinases (MMPs) are a family of proteases that degrade structural components of the ECM. Although first recognized for their role in amphibian metamorphosis, ^[2]^ MMP-mediated tissue remodeling is known to regulate branching morphogenesis and wound healing, and is increasingly recognized as fundamental for creating tissues for therapeutic applications. ^[3]^ MMPs have also been implicated in diverse pathological processes ranging from aortic aneurysm, ^[4]^ to chronic obstructive pulmonary disease, ^[5]^ and the epithelial to mesenchymal transition, a known driver of tumor malignancy. ^[6]^

Despite their importance and ubiquity, the precise role MMPs play in myriad biological contexts is incompletely understood. Knock-out animal models produce surprisingly subtle phenotypes, likely because of enzymatic redundancies, compensation and adaptive development, ^[7]^ and stained tissue sections provide only limited information on MMP-mediated tissue dynamics. To address these limitations, zymography can measure MMP proteolytic activity, however, this electrophoretic technique cannot provide *in situ* information, and although quantitative, can separate tissue inhibitors of metalloproteinases from their bound MMP, leading to unnatural enzyme activity. ^[8]^ Enzyme-linked immunosorbent assay-based methods are similarly quantitative, but again do not provide *in situ* information. The same drawbacks limit the utility of voltametric-based methods that rely on MMP activity to expose electrodes, making them available for redox mediator conjugation. ^[9]^ Surface plasmon techniques, ^[10, 11]^ which exploit the change in refractive index (and thus the resonance) upon substrate proteolysis, are quantitative, and can provide a local measurement by using probes that bind covalently at an enzyme’s catalytic sites *in situ*. ^[12]^ However, as the probe itself nullifies enzymatic activity, this approach precludes the study of real-time enzyme activity dynamics.

Probes capable of combining MMP substrates with fluorescent molecules have emerged as real-time monitors of enzymatic activity, and include genetically encoded fluorescent proteins, peptides labelled with small molecule dyes, quantum dots, and other nanoparticles. ^[13]^ Förster resonance energy transfer (FRET) probes designed such that FRET efficiency is altered by substrate cleavage are a promising tool. Here, when the FRET pair are held in close proximity, energy of the donor fluorophore is transferred to the acceptor. However, upon MMP-mediated cleavage, the donor’s unquenched fluorescence is observed, providing a quantitative real-time and *in situ* measurement of enzyme activity.

Intensity-based fluorescence measurements are often used to monitor FRET; however, the differing acceptor to donor stoichiometry at any location needs to be compensated for during analysis, as intensity-based FRET measurement are fluorophore-concentration dependent. Alternatively, fluorescence lifetime imaging (FLIM), ^[14]^ which excites fluorophores using a pulsed laser and monitors the time-dependent fluorescence decay, provides concentration-independent and highly accurate measurements of FRET efficiency. Fluorescence lifetime is insensitive to variations in excitation light intensity, detector response, and donor photobleaching. And, unlike intensity-based FRET measurements, lifetime is not impacted by spectral bleed-through or direct acceptor excitation, which can confound interpretation ^[15, 16]^. FLIM measurements of FRET have proven immensely powerful in understanding complex intracellular processes. For example, such techniques have identified the direct negative regulator of actin related protein 2/3 complex-mediated lamellipodia formation during mesenchymal cell migration ^[17]^. They have also been used to study how multiple conformations of cardiac myosin binding protein-C bind to thick and thin filaments during heart muscle contraction ^[18]^.

Engineered tissues formed by combining cells with biomaterial scaffolds offer the possibility of studying tissue formation and cell-ECM interactions, as well as modelling physiological and pathological processes, all within reductionist 3D environments amenable to biological and physical control. Biocompatible fully synthetic polyethylene glycol (PEG)-based hydrogels formed using thiol-ene click chemistries ^[19–21]^ can mimic many properties of the native ECM, ^[22, 23]^ and are widely used in these applications. To provide a more native-like environment, peptides that encode integrin-binding domains can be included, and hydrogels cross-linked with peptides susceptible to degradation by MMPs. ^[24]^ Thus, as cells produce MMPs, they remodel their surroundings, providing a microenvironment that mimics many aspects of matrix remodeling within native tissues.

Here, we incorporate a FRET sensor of MMP activity into PEG-based hydrogels. We then use FLIM measurements of FRET to quantify MMP activity in these native tissue-like environments, establishing a highly accurate platform for quantitatively studying MMP dynamics *in situ*. Encapsulation of MCF7 human breast cancer cells within these hydrogels, further shows the ability of the platform to detect cell-derived MMP activity both locally, at the sub-micron level, and within the bulk hydrogel. Our versatile platform may find use in a range of biological studies to explore questions in cancer metastasis, development, and tissue repair.

## 2 Results

### 2.1 FRET protein design, purification and characterization

**Figure 1:**
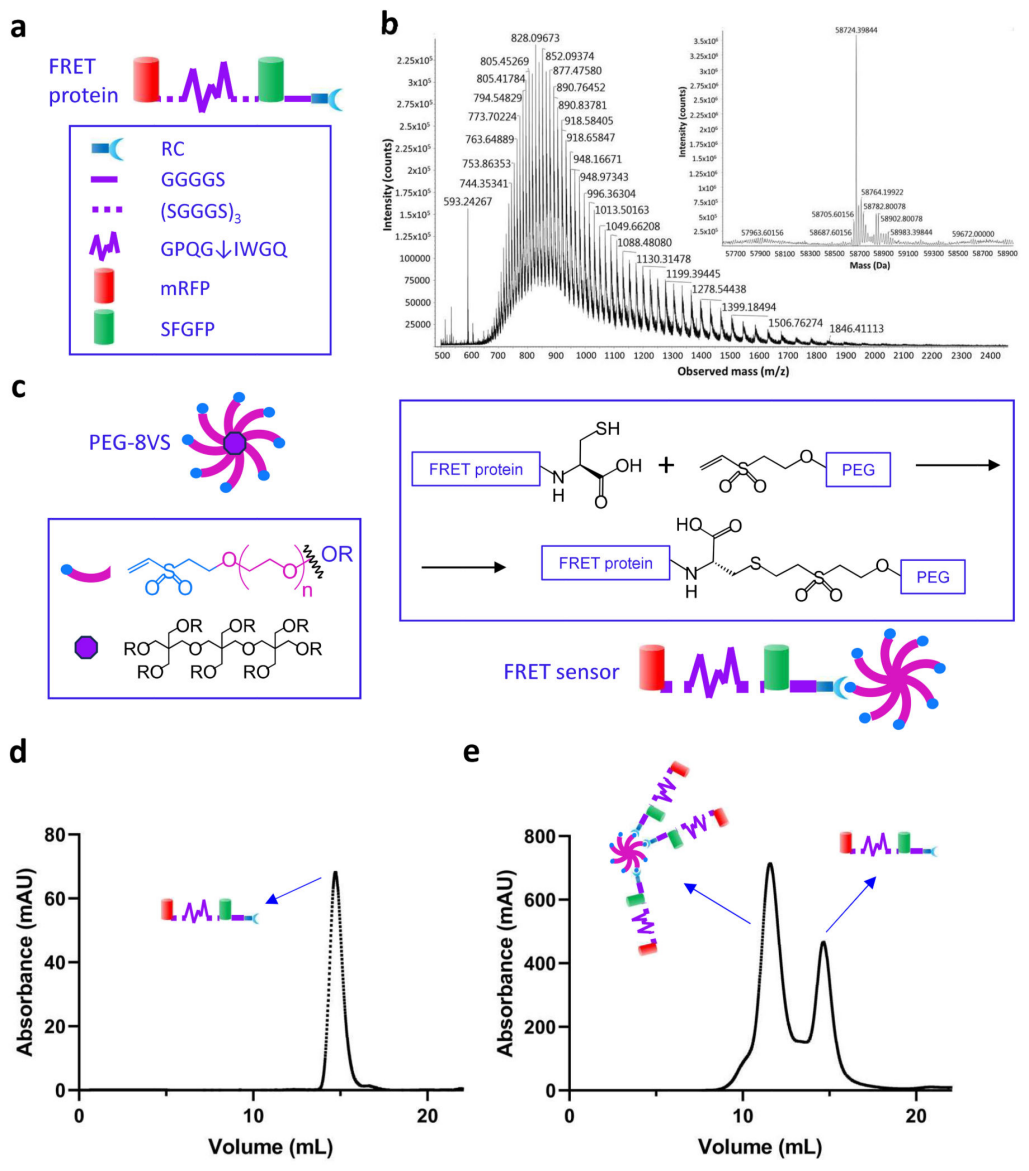
**a**. Design of the FRET protein, which includes an MMP-degradable peptide sequence (GPQG↓IWGQ) flanked by repeating linker sequences (SGGGS)_3_ placed between SFGRP (donor) and mRFP (acceptor). An additional linker sequence after the SFGFP separates it from an arginine and then a cysteine at the C-terminal. ↓ indicates the enzymatic cleavage site in the peptide. **b**. Mass spectrometry of the FRET protein, with inset showing the deconvoluted spectrum. The theoretical mass of the protein based on its amino acid sequence and after cyclization correction was 58726.27 Da, which matched that of the deconvoluted mass identified in the spectrum (58724.39 Da). **c**. Schematic showing the conjugation of the FRET protein to PEG-8VS. The FRET protein is conjugated to the PEG via a covalent bond between the thiol on the C-terminal cysteine and vinyl sulfone (VS) to create the FRET sensor. **d**. SEC elution spectra of FRET protein, and **e**. PEG-FRET protein conjugation product (FRET sensor) after 1 h incubation. Eluted peaks at 11.51 and 14.67 mL correspond to PEG molecules conjugated with 3-4 FRET proteins and unconjugated FRET proteins, respectively.

We first designed both a FRET protein consisting of a monomeric red fluorescent protein (mRFP) ^[25, 26]^ (acceptor), a peptide, and a cysteine-free green fluorescent protein (GFP) variant (donor, SFGFP) joined in sequence, as well as a donor-only protein (Figure 1a, Tables S1 and S2). The peptide included an MMP-susceptible cleavage site flanked by a repeated linker sequence (SGGGS) to ensure accessibility of the construct to MMPs. To enable conjugation of the protein to vinyl sulfone (VS)-functionalized PEG molecules using thiol-ene click chemistry, we also placed a cysteine residue after the C-terminal sequence of SFGFP and spatially separated it from the MMP cleavage site using a linker sequence (GGGGS) and an arginine residue. Here, arginine creates a cationic local environment to foster deprotonation of the cysteine thiol group, and thus promote the reaction with VS. As neither SFGFP nor mRFP contain endogenous cysteine residues, VS-mediated interactions with the fluorescent protein are precluded. The donor-only protein’s sequence was designed identically to that of the FRET protein, except it did not contain mRFP.

After cloning and sequencing, we overexpressed the FRET and donor-only proteins in *E. coli*. Crude protein was extracted and purified using affinity and size exclusion chromatography (SEC). Analysis using sodium dodecyl-sulfate polyacrylamide gel electrophoresis (SDS-PAGE) yielded bands at the proteins’ theoretical molecular weights of 58 kDa (FRET protein) and 33 kDa (donor-only protein) and showed better than 95% purity (Figure S1a). This was further confirmed using a calibration based on proteins with known molecular weights to predict the proteins’ elution volumes using SEC (Figure S1b-e). The proteins were also analyzed by mass spectrometry (Figure 1b and Figure S2). Spectra show that the deconvoluted mass of both the FRET protein and donor-only protein matched the corrected theoretical masses of the respective proteins. Taken together, these data confirm the successful production and purification of the FRET and donor-only proteins.

### 2.2 FRET protein tethered to PEG monomers shows good FRET efficiency

To tether the FRET (or donor-only) protein into a synthetic hydrogel, we optimized its conjugation to 8-arm PEG-VS (PEG-8VS) through a Michael addition between the cysteine residue of the protein and VS (Figure 1c and Figure S3a). The multi-arm PEG offers the opportunity to tether up to 8 FRET proteins per molecule; however, as PEG-8VS also mediates hydrogel cross-linking, we aimed to limit the number of arms conjugated to the protein. We calculated the theoretical elution volumes of the eight possible conjugation products (Table S3), and then tuned the reaction time and the stoichiometry before assessing the output by SEC (Figure 1d-e). For a reaction time of 1 h and a stoichiometric ratio of 12.5:1 (PEG-8VS to FRET protein), we identified a peak at an elution volume of 11.57 mL, corresponding to PEG-8VS conjugated with 3 or 4 FRET proteins. The FRET-protein-PEG conjugates from this elution is hereafter referred to as the “FRET sensor” and the corresponding donor-only protein conjugation product as the “donor-only control”.

**Figure 2:**
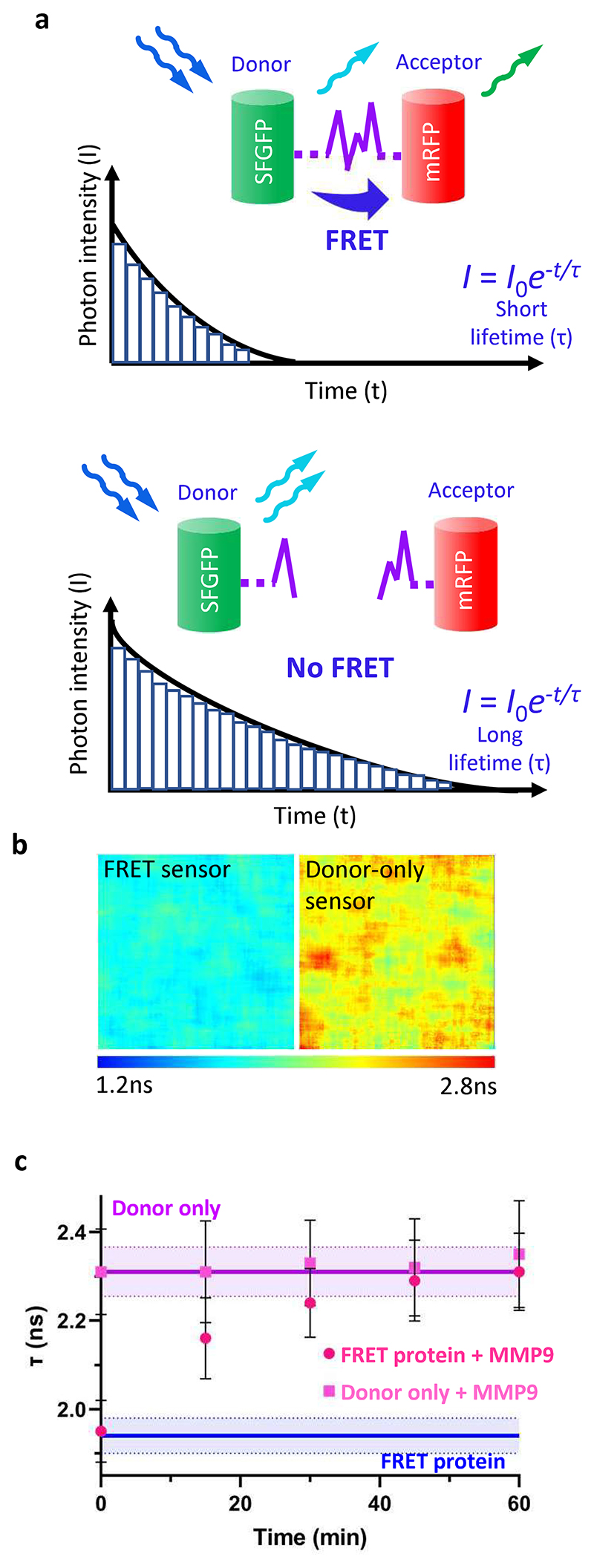
**a**. Schematic illustrating impact of FRET on fluorescence lifetime measurements. **b**. Example heat maps showing donor lifetime (τ) generated from FLIM measurements on the donor-only (SFGFP) control and the FRET sensor. Red colors indicate longer lifetimes in the absence of FRET, while blue colors show shorter lifetimes when FRET is active. **c**. Donor lifetime, τ (mean ± S.D.) of the FRET protein and donor-only protein control treated with 35 nM MMP9 measured over 60 min. The lifetime of the FRET protein and donor-only protein control in the absence of MMP9 treatment are shown for comparison (mean ± S.D., shaded area). n=3, for all groups.

Fluorescence lifetime measurements rely on exciting fluorophores with a pulsed laser and then quantifying the time they remain in their excited state before returning to their ground state through release of a photon. ^[27, 28]^ In the presence of FRET, the fluorescence lifetime (τ) of the excited donor is shortened since energy is transferred to the acceptor (Figure 2a). To test the FRET efficiency of our FRET sensor in solution, we first measured τ of its SFGFP component, which we compared to τ of SFGFP component within the donor-only control. SFGFP was imaged for 300 s, and photons collected from each pixel. Photon arrival times were then plotted as histograms and fitted with a mono-exponential decay curve to determine the average lifetime per pixel. This allowed us to represent lifetime as a color heat map, where longer lifetime is red and shorter is blue (Figure 2b). We found that compared to the donor-only control, the FRET sensor had a τ that was significantly shorter (FRET sensor (mean±s.d.): τ = 1.88±0.06 ns; donor-only control: τ = 2.35±0.13 ns; *p* < 0.01, unpaired two-tailed t-test, *n* = 3). Based on these measurements, the FRET efficiency was calculated to be 20.25±5.17%, which was within the expected range for these fluorophores. ^[29]^

### 2.3 FRET protein responds to MMP treatment as a change in fluorescence lifetime

To confirm the functionality of the FRET protein in terms of its ability to respond to MMP-mediated cleavage via a change in fluorescence lifetime, we next treated it and the donor-only control with 35 nM MMP9 and measured τ. Fluorescence lifetime of the MMP9-treated FRET protein was significantly longer than that of the untreated FRET protein after 15 min (Figure 2c and Figure S4)(*p* = 0.02, unpaired two-tailed t-test). After 30 min, τ of the FRET protein treated with MMP9 was no different than that of the donor-only protein, confirming loss of FRET after MMP-mediated cleavage of the peptide. We also verified the ability of the FRET sensor to respond to MMP-mediated peptide cleavage using standard measurements of fluorescence intensity. To accomplish this, we excited SFGFP and quantified fluorescence intensity at 610 nm, the peak of the emission spectrum of mRFP, and found that it was significantly lower in MMP9-treated samples compared to untreated FRET sensor controls, confirming loss of FRET (Figure S5). Taken together, these data confirm that MMP-mediated cleavage of our engineered system could be monitored by measuring a change in fluorescence lifetime or fluorescence intensity.

### 2.4 FRET-sensor modified hydrogels respond to exogenous MMP

**Figure 3:**
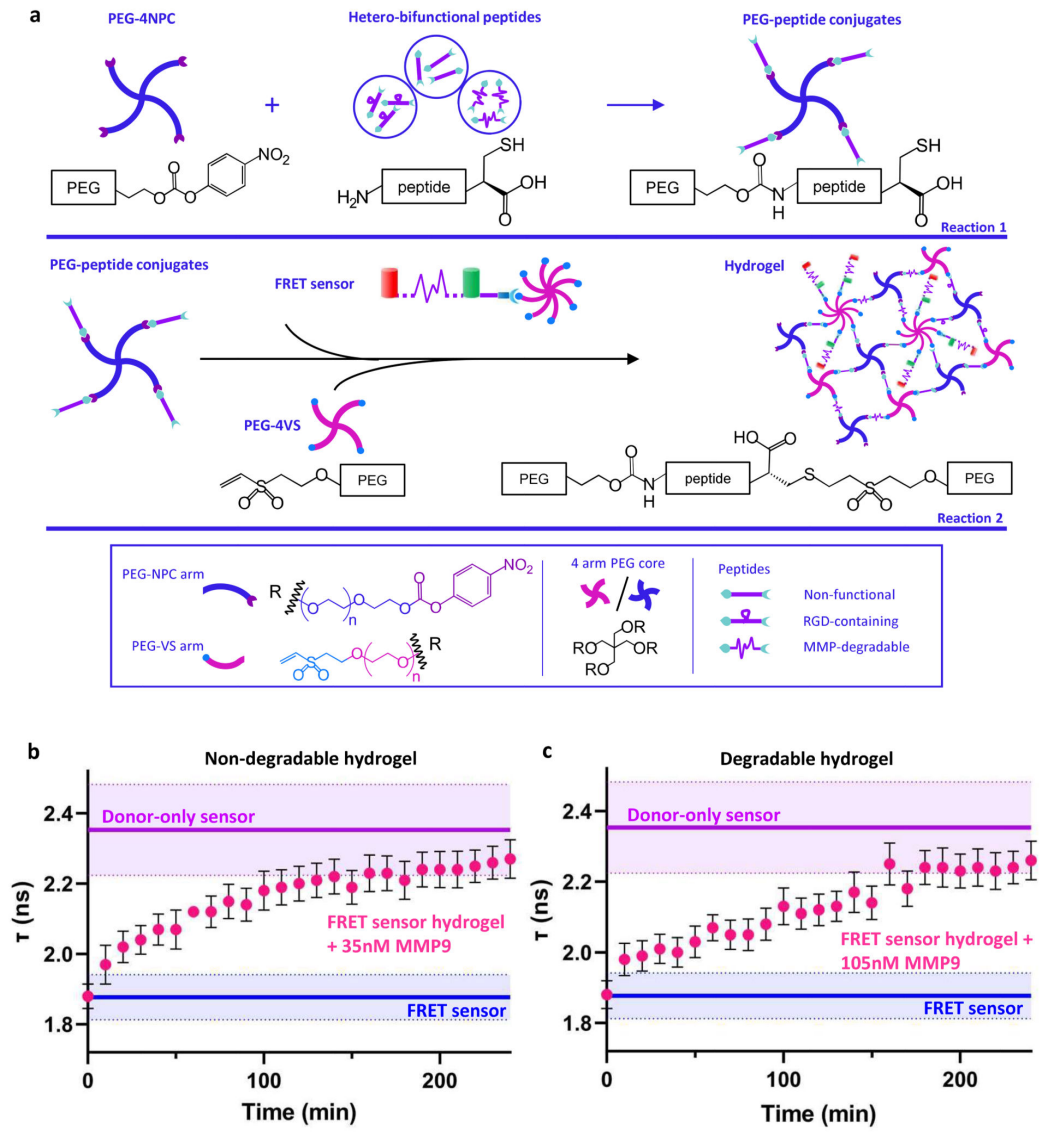
**a**. PEG hydrogel design incorporating the FRET sensor. PEG-peptide conjugates are formed by reacting the chain-end carbonate groups of PEG-NPC with primary amines on N-terminal lysines, forming stable carbamate bonds between PEG arms and peptides (Reaction 1). PEG-peptide conjugates are then mixed with PEG-4VS and FRET sensor, which both contain PEG arms functionalized with vinyl sulfone (VS) at the chain end (Reaction 2). The Michael addition between VS and the cysteine's thiol on PEG-peptide conjugates generates cross-links leading to hydrogel formation. **b**. Non-degradable hydrogels and **c**. degradable hydrogels, both containing the FRET sensor, were treated with 35 nM and 105 nM MMP9, respectively. Plots show the FRET sensor lifetime (mean τ ±S.D., shaded area), as a function of time. Soluble FRET sensor and donor-only sensor are shown for comparison (Donor-only sensor, FRET sensor n=3; FRET sensor hydrogel+MMP9 n=4)

To create hydrogels containing the FRET sensor, we built from our existing design, which relies on a two-step conjugation reaction. ^[20, 30]^ Heterobifunctional peptides that are either non-functional or contain an MMP-degradable or an RGD sequence are first conjugated to 4-arm PEG functionalized at each terminus with nitrophenyl carbonate through a nucleophilic substitution reaction with the primary amine on an N-terminal lysine (Figure 3a). The network is cross-linked through a Michael addition between a C-terminal cysteine on the peptide with PEG-4VS in a 1:1 stoichiometric ratio. To create hydrogels containing the FRET sensor, 1 μM PEG-4VS was replaced with 1 μM of FRET sensor (or donor-only control) (Figure 3b). Within each FRET sensor, three to four arms are conjugated with FRET proteins, leaving the remaining four to five arms available for cross-linking. Thus, the total number of VS groups available for cross-linking remains unchanged, and stable hydrogels form similarly to those that do not contain the FRET sensor. Measurements of hydrogel gelation by oscillatory rheology confirmed that the mean plateau modulus (G’) of FRET sensor-containing hydrogels was no different (*p* = 0.12) than that of standard hydrogels without the FRET sensor (Figure S6), confirming that incorporation of the FRET sensor did not impact hydrogel mechanical properties.

We next aimed to determine if the FRET sensor was still responsive to MMP-mediated cleavage upon incorporation into PEG hydrogels. To avoid competition for MMP activity between MMP-sensitive peptides that act as hydrogel cross-linkers and the FRET sensor, we first cross-linked hydrogels with peptides that were not susceptible to degradation by MMPs (non-degradable/adhesive) (Table S4a). We then monitored fluorescence lifetime over 240 min after incubating FRET sensor-modified hydrogels with 35 nM MMP9. Fluorescence lifetime in hydrogels treated with MMP9 was no different than that in the FRET sensor control at time 0 and 10 min; however, after 20 min of treatment, τ in was significantly longer (*p* = 0.0174, unpaired two-tailed t-test) and continued to increase (Figure 3c and Figure S7a). After 100 min, τ in hydrogels was no different (*p* > 0.05) than that in the donor-only control. Measurements of acceptor-to-donor (mRFP:SFGFP) fluorescence intensity ratios similarly confirmed a significant decrease after 4 h of MMP9 treatment (Figure S8). Taken together, these data suggest that that the FRET sensors in the hydrogel had been fully cleaved by the enzyme. They also demonstrate that the fluorescence lifetime of the FRET sensor was still measurable when incorporated into a PEG hydrogel, and that it was possible to measure enzymatic cleavage of the sensor as a change of fluorescence intensity or lifetime within the hydrogel.

### 2.5 FRET sensor still detects protease activity in MMP-susceptible hydrogels

Synthetic hydrogels are often designed to be covalently cross-linked with MMP-degradable peptides. This allows encapsulated cells to actively remodel their surroundings, enabling changes in morphology, ^[31]^ matrix secretion, ^[3, 32]^ and growth of multicellular structures like organoids. ^[20, 33]^ Therefore, we next asked if the FRET sensor was still responsive to MMPs when tethered within an MMP-degradable hydrogel that would simultaneously compete with the sensor for enzyme. In this context, the hydrogel itself contains 1211.4 μM MMP-degradable peptide (degradable/adhesive, Table S4b), or 303 times more MMP-cleavable sequences than the tethered FRET sensors themselves. Despite this, we were still able to monitor similar MMP-mediated changes in lifetime to those we observed in the non-degradable hydrogel (Figure 3d and Figure S7b), with full cleavage of the FRET sensor (no different than the donor-only control, *p* > 0.05, unpaired two-tailed t-test) requiring 160 min when treated with 105 nM MMP9.

Monitoring of lifetime in both degradable and non-degradable FRET-sensor-containing hydrogels in the absence of MMP9 treatment never produced values for τ that were different from those of the FRET sensor-containing hydrogel alone (Figure S9a), confirming the specificity of changes in τ to MMP activity. Although achieving fluorescence lifetime values that were significantly different (*p* = 0.0375) to those of the FRET sensor within 20 min required raising MMP levels from 35 nM to 105 nM (Figure 3d and Figure S9b), this finding demonstrates the exquisite sensitivity of the FLIM system for monitoring FRET as a read out of MMP activity within complex 3D systems suitable for studying matrix remodeling *in vitro*.

### 2.6 FRET sensor-modified hydrogels detect MMPs produced by encapsulated cells

**Figure 4:**
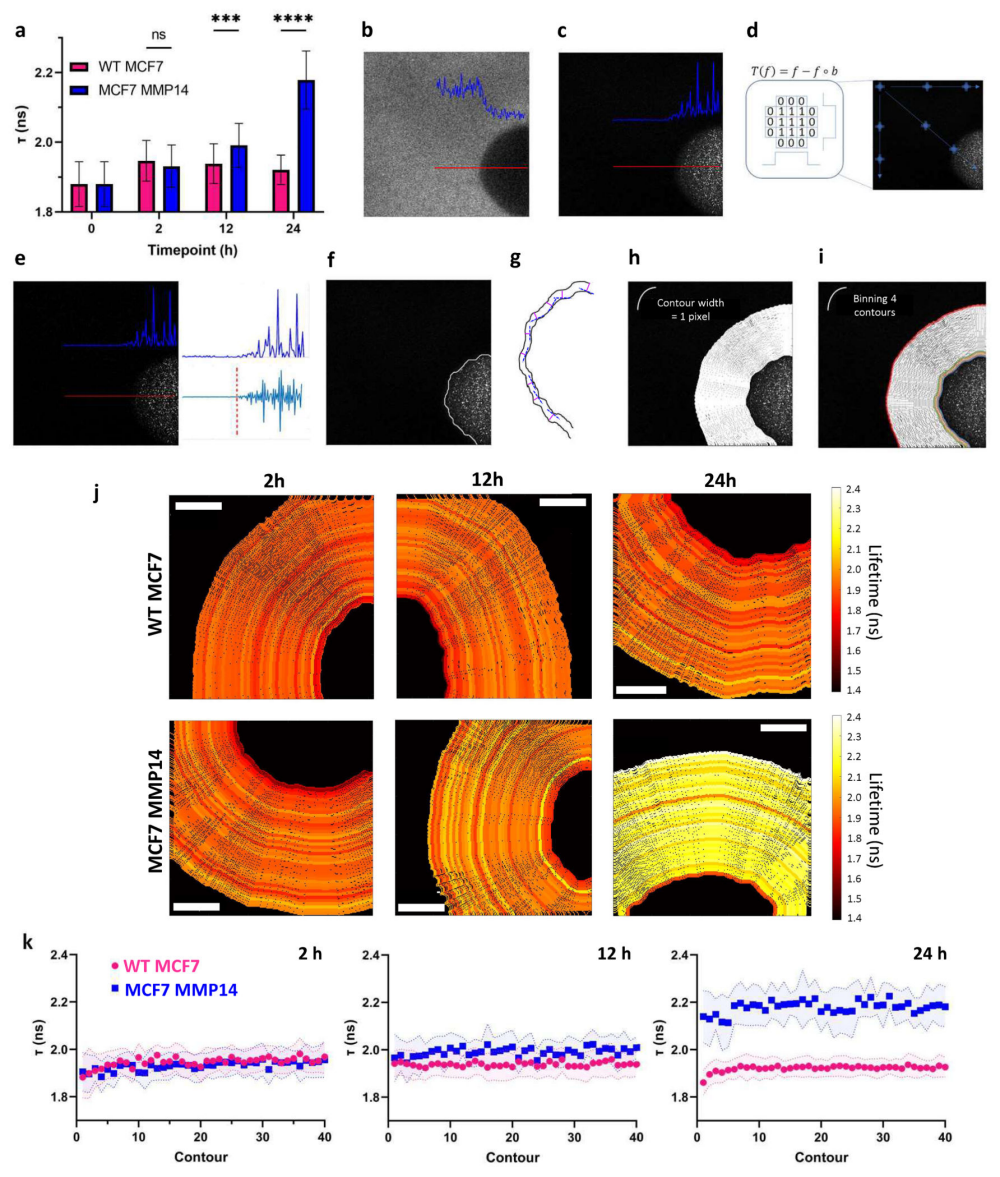
**a**. Mean τ (± S.D.) around WT MCF7 and MCF7 MMP14 cells collected by pooling 40 contours around each cell (***p<0.001; ****p<0.0001; unpaired two-tailed t-test). Workflow to analyze fluorescence lifetime (τ) around a cell: **b**. A raw FLIM image is **c**. inverted and normalized to the maximum intensity value. **d**. The image is filtered using a top hat strategy, followed by **e**. a Canny edge detection strategy to identify the edge of the cell. **f**. Based on these analyses, a primary contour is drawn. Then, by **g**. constructing a unit normal (magenta line) to the tangent (blue dashed line), **h**. additional 1 pixel contours are propagated equidistant to the initial contour. **i**. To avoid oversampling of a laser spots size, which was 4.73 pixels, 4 layers of pixels (contours) were binned. **j**. Example heat maps showing contours of fluorescence lifetime (τ) extending radially from the cell, which appears black. Scale bar= 5 μm. **k**. Fluorescence lifetime (τ) (mean ± S.D., shaded area) as a function of contour number extending from membrane of WT MCF7 and MMP14 MCF7 cells encapsulated within hydrogels. (in **a** and **k**, *n* varied between 11 and 33 cells for all groups).

Having established our system’s responsiveness to exogenous MMPs, we next aimed to test the ability of the FRET sensor-modified hydrogels to monitor MMP activity by cells encapsulated with them. We encapsulated the human breast cancer cell line MCF7 in non-degradable/adhesive hydrogels and confirmed that they remained viable after up to 5 days in culture (Figure S10). Wild type MCF7 (WT MCF7) cells produce only negligible levels of MMPs; ^[34, 35]^ however, a cell line based on MCF7 that stably expresses MMP14 (MCF7 MMP14), has been shown to degrade ECM components. ^[36]^ Therefore, we encapsulated both WT MCF7 and MCF7 MMP14 cells in non-degradable/adhesive hydrogels and monitored τ after 2, 12 and 24 h and found that fluorescence lifetime was significantly longer in hydrogels containing the MCF7 MMP14 cells than in hydrogels containing the WT MCF7 cells after both 12 and 24 h of culture (Figure 4a). These findings confirm that encapsulated cells can produce MMPs that cleave the FRET sensor, which can be monitored over time as a change in fluorescence lifetime.

### 2.7 Cells within PEG hydrogels do not produce local gradients of MMP activity

Cell-produced MMPs can either be secreted or remain membrane-bound. Therefore, a reasonable hypothesis is that MMP activity would be higher closer to cells compared to farther away, resulting in local stiffness gradients. However, counter-intuitively, in synthetic hydrogels, passive micro-rheology measurements suggest that MMP-mediated hydrogel degradation by encapsulated human mesenchymal stem cells occurs to a greater extent at distances farther from cells compared to closer. ^[37]^ Optical tweezer-based measurements similarly show no difference in hydrogel stiffness in areas close to encapsulated cells compared to areas that are farther away. ^[38]^ However, both these methods rely on measurements using micro-beads, limiting their resolution. Therefore, we next aimed to develop a pipeline to quantify local FRET activity by measuring fluorescence lifetime within contours drawn at the level of single pixels around individual cells.

FLIM imaging around individual cells was performed by exciting SFGFP at 920 nm, yielding images in which the hydrogel appears bright and encapsulated cells are dark (Figure 4b). To identify the position of the cell membrane, we produced custom MATLAB code (Supplementary Code 1) that first uses a top hat filtering strategy to enhance the signal-to-background ratio. We then applied Canny edge detection techniques, ^[39]^ to allow for precise edge detection, from which we drew a primary contour (Figure 4c-e). This primary contour was then used to propagate subsequent contours normal to the tangent of and equidistant from the primary contour at every pixel (Figure 4f-g). This process was repeated to create a continuum of contours, which we then binned into groups of 4 in accordance with the laser spot size (4.7 pixels), to avoid oversampling (Figure 4h-i).

We then encapsulated WT MCF7 and MMP14 MCF7 cells in non-degradable/adhesive hydrogels and measured τ in contours around encapsulated cells, which we visualized as heat maps (Figure 4j). In keeping with bulk measurements of τ, heat maps of contours around MMP14 MCF7 cells showed longer fluorescence lifetimes after 12 and 24 h compared to heatmaps around WT MCF7 cells, whose τ remained stable. We then plotted τ as a function of the number of contours extending from cells (Figure 4k), but regression analysis could not detect significant differences in the slopes of the curves when comparing the WT MCF7 to MMP14 MCF7 cells (*p* > 0.05). This suggests that there was no preferential degradation of the FRET sensor closer to the cell membrane compared to further away in MMP14 MCF7 cells compared to WT MCF7 cells, within the range we measured.

## 3 Discussion

Here, we describe a FRET sensor, which we incorporate into synthetic hydrogels to monitor MMP activity, creating an *in vitro* platform capable of monitoring cell-mediated matrix remodeling in native tissue-like 3D environments. Our FRET sensor is based on mRFP-mediated energy quenching of SFGFP, a coupling which remained effective even upon conjugation of the protein to PEG monomers. We then tethered our sensor into PEG hydrogels that have previously been shown to support mesenchymal remodeling around human iPSC-derived intestinal organoids. ^[20]^ Our FRET sensor-containing hydrogels responded to treatment with exogenous MMPs, which prompted increases in fluorescence lifetime, irrespective of the hydrogel’s own susceptibility to degradation by MMPs. We then encapsulated an MMP-expressing human breast cancer cell line within hydrogels and demonstrated real-time monitoring of cell-produced MMP activity both at the bulk and local levels around cells. Taken together, these data demonstrate that our system can monitor matrix remodeling by live cells *in situ* and in real time within physiologically relevant 3D tissue models.

MMP-susceptible FRET sensors have previously been incorporated into PEG hydrogel microbeads, which were incorporated into bulk unmodified hydrogels. ^[40]^ Using this approach, the authors showed gradients of MMP activity extending out from cancer cell spheroids within the bulk hydrogel. However, microbead-based strategies provide relatively low resolution of enzymatic activity within hydrogels. In contrast and in keeping with recent reports, ^[37, 38]^ we found that MMP activity was not higher closer to individual cells compared to farther away. This is counter-intuitive as native MMP activity, like that of many morphogens, is regulated locally. ^[41]^ PEG hydrogels mimic many aspects of the native ECM; however, they are imperfect substitutes. Because they lack vasculature, hydrogels are designed to permit the diffusion of small molecules and proteins. Indeed, the permeability of hydrogels can be orders of magnitude higher than that of native tissues. ^[42]^ Thus, while MMPs can be relatively large (MMP9: 92kDa, MMP2: 72kDa), mathematical modelling and experimental measurements show that within low polymer concentration hydrogels, even large molecules diffuse at similar rates to small molecules. ^[21, 43]^ This suggests that although our system is capable of capturing local MMP activity around cells, *in vivo*-like gradients are not established in these highly permeable materials. It is possible to tune hydrogels by increasing polymer concentration to reduce mesh size and thus limit MMP diffusion; however, highly dense hydrogels confine cells and can fail to support viability. ^[44]^ This remains a limitation of current designs, but one that might be addressed by incorporating microfluidic channels or other nutrient delivery approaches into dense materials with diffusive properties that match those of native tissues.

We created our MMP activity sensor by genetically encoding an MMP-cleavable peptide sequence between FRET proteins. Using this approach, the enzyme cleavage site can be tailored through mutagenesis, allowing the broadly susceptible sequence we used here to be replaced by sequences recognized by specific MMPs. For example, patients with inflammatory bowel disease show upregulation of MMP9 in their inflamed intestinal tissue, ^[45]^ which may contribute to fibrotic remodeling of the gut mesenchyme; ^[46]^ and MMP3 plays key roles in branching morphogenesis in tissues such as the breast. ^[47]^ Thus, targeted modification would allow for mapping of specific MMPs’ activities around encapsulated cells/organoids/explants with sub-cellular resolution. Further applications of our platform might include building organoid-based models to study the role of MMPs in pulmonary fibrosis, exploring the roles of immune cell-derived MMPs in cancer metastasis, or studying fundamental processes that drive branching morphogenesis in tissues such as salivary glands or the kidney. Moreover, the versatility of the approach is such that different fluorescent proteins, and thus FRET pairs, can be incorporated, which would allow for spectral multiplexing and potential co-localization studies of multiple MMPs. Recent reports have shown that cells and organoids encapsulated within hydrogels can remodel their surroundings through pericellular/peri-organoid ECM secretion. ^[32, 48, 49]^ Thus, our sensor, could find use in mapping the dynamics of pericellular matrix remodeling, which may reveal insight into how the secreted matrix can be harnessed to create tissues, particularly for regenerative applications.

Our approach for incorporating the FRET sensor into biomaterials using a cysteine’s thiol group should allow for its use in a range of synthetic and biologically derived hydrogels or cell-derived ECMs (Matrigel, *e.g*.) with little to no additional modification. This is in contrast to reports of a collagenase-susceptible fluorescent peptide that could be incorporated into a collagen hydrogel, but which required a series of complex chemical modifications. ^[50]^ Moreover, our approach avoids genetically encoding the MMP sensor directly into target cells, ^[51–53]^ which often requires high levels of intracellular overexpression to obtain accurate measurements of MMP activity using FRET. ^[54]^ Thus, our approach should allow for monitoring of MMP activity around cells and multicellular aggregates, including those that are difficult to genetically modify, such as explants, organoids, and assembloids. However, despite these advantages, our FLIM-based approach may not be suitable in some applications. Indeed, as we rely on time correlated single photon counting, the accuracy of the measurement is strongly influenced by the number of detected photons ^[55]^, making the time scale of the measurement relatively long. Thus, imaging scenarios requiring quick acquisition may not be amenable to the technique.

To our knowledge, this is the first report that uses FLIM/FRET to monitor MMP activity within 3D engineered microenvironments. Previous work that incorporated collagenase-susceptible biosensors into collagen hydrogels, ^[50]^ and MMP-sensitive biosensors into PEG hydrogels ^[40]^ both relied on using fluorescence intensity to quantify enzyme activity. However, as fluorescence intensity measurements are fluorophore concentration-dependent, inhomogeneities can complicate and even skew calculations, and extensive background fluorescence intensity normalization is required to ensure data accuracy ^[15]^. By employing FLIM, our approach precludes such issues, ensuring highly accurate measurements at subcellular resolution. Since the FLIM measurement is independent of sensor concentration, our FRET sensor does not suffer from these drawbacks. Moreover, FLIM is becoming more accessible as standard confocal laser scanning microscopes can be upgraded with relatively straightforward add-on kits that allow for measurements of lifetime without the need for complex custom-built setups (see picoquant.com, *e.g*.). Thus, the platform we describe here is versatile, easily modifiable for different biological applications, and accessible to standard laboratories.

## 4 Conclusions

Many human pathologies are driven by aberrant matrix remodeling. Our MMP-sensitive FRET sensor hydrogel may allow for monitoring of matrix remodeling, as well as mechanistic experiments aiming to unravel how MMP activity contributes to both normal and pathological cell behaviours. Indeed, our platform has the potential to provide novel insights into cancer metastasis, physiological tissue dynamics, and pathological matrix remodeling, by providing both unparalleled sub-cellular resolution of real-time MMP activity and quantitative analytical readouts.

## 5 Experimental section

### Plasmid construction

#### pET151 FRET protein plasmid

A DNA oligo fragment containing the MMP-cleavable peptide (GPQG↓IWGQ, where ↓ indicates the cleavage site), linkers, cysteine-free superfolding GFP (SFGFP), and the two additional amino acid residues (arginine and cysteine) was purchased from Integrated DNA Technologies. Primers were designed using NEBuilder Assembly Tool 2.0 and the DNA oligo was amplified via polymerase chain reaction (PCR) using Q5 High-Fidelity DNA Polymerase (NEB M0491), creating the cloning insert (Cloning Insert forward: 5’ - atgggcataaattctcggtccgcggtgaaggtgagggg - 3'; Cloning Insert reverse: 5’ - gatctgagctcgcccttttaacagcgacttccaccacc - 3'). A pET151 RFP-GFP plasmid ^[26]^ (a gift from the Beavil Group, King’s College London) was also amplified by PCR, creating the cloning vector (Cloning Vector forward: 5’ - taataagggcgagctcagatccgg - 3'; Cloning Vector reverse: 5’ - gaccgagaatttatgcccatttacatcaccatc - 3'). The cloning insert and vector were then ligated with NEBuilder HiFi DNA Assembly Master Mix (NEB E2621), and the product transformed into *Escherichia coli* XL-1 blue strains (Agilent 200249) to produce the pET151 FRET protein plasmid. Transform-positive XL-1 blue cells were selected using LB agar plates containing 100 mg mL^-1^ ampicillin. Once selected, XL-1 blue colonies were picked by hand and grown in LB medium with 100 mg mL^-1^ ampicillin for 16 h at 37 °C. Plasmids were purified using the QIAprep Spin Miniprep kit (Qiagen 27104), and their sequences were analyzed by Source Bioscience Ltd. The FRET protein encoding sequence is shown in Table S1.

#### pET151 donor-only protein plasmid

A DNA oligo fragment containing the encoding sequence for SFGFP, linkers and two amino acid residues (arginine and cysteine) was purchased from Integrated DNA Technologies. The PCR amplified product of this oligo (Cloning Insert forward: 5’ - gatataccatgggcagcagccatcatcaccatcaccatg-3'; Cloning Insert reverse: 5’ – tgttagcagccggatccttattaacagcgacttccacc - 3') was then ligated with the PCR amplified product of the pET151 RFP-GFP plasmid (Cloning Vector forward: 5’ - taaggatccggctgctaac-3'; Cloning Vector reverse: 5’ – gctgctgcccatggtatatc - 3'). The pET151 donor-only protein plasmid was then produced identically to that FRET protein plasmid (as above). The sequence of purified plasmid was analyzed by Source Bioscience Ltd. The donor-only protein encoding sequence is shown in Table S2.

### Protein expression, purification and characterization

Plasmids containing the encoding sequences for the FRET and donor-only proteins were transformed into *Escherichia coli* BL21(DE3) cells (Agilent 200249). Transfected BL21(DE3) cells were cultured in 10 mL LB media with 100 mg mL^-1^ ampicillin at 37 °C for 16 h. The resulting 10 mL pre-culture was then added to 250 mL auto-induction media ^[56]^ with 100 mg mL^-1^ ampicillin and cultured at 16 °C for 72 h. Pellets were harvested via centrifugation at 12,000 g for 20 min and lysed using the BugBuster Mater Mix (Merck 71456). The supernatant of the auto-induction bacteria lysate was then harvested by centrifugation at 12,000 g for 20 min and purified by affinity chromatography using HisTrap HP columns (GE 175248), and then size exclusion chromatography (SEC) using Superdex columns (GE 28-9909-44). Affinity chromatography was performed in 25 mM HEPES (pH 8) with 150 mM NaCl and 300 mM Imidazole at room temperature (RT) to elute the polyhistidine-tagged proteins. The SEC buffer was composed of 25 mM HEPES (pH 8) with 150 mM NaCl. SEC purification was performed at 4 °C in 25 mM HEPES (pH 8) with 150 mM NaCl at 0.75 mL min^-1^ over 1 column volume. A Gilson HPLC system was paired with the SEC for sample collection. Final protein products were analyzed by SDS-Polyacrylamide Gel Electrophoresis (PAGE) using NuPAGE Bis-Tris protein gels (Thermo Fisher NP0322). After staining with Coomassie blue (Abcam ab119211) for 15 min, SDS-PAGE gels were visualized using a ChemiDoc XRS+ (Bio-rad). Band intensities were calculated in Image J, and the intensity of target protein band was compared to the relative intensity of all protein bands to calculate purity. High resolution mass spectrometry was also performed to confirm the homogeneity of protein products using a Waters Xevo G2-XS QTof. Mass spectrometry was run in MS^E^ fragmentation mode with electrospray ionization using the following settings: positive polarity, capillary voltage 3.0 kV, source temperature 80 °C, sampling cone 30 V, desolvation temperature 200 °C, m/z range 50–3000, and transfer collision energy ramp 25-45 V.

### Calculation of theoretical elution volume of the FRET protein and donor-only protein

Five standard protein markers with known MW (thyroglobulin 669 kDa, apoferritin 443 kDa, alcohol dehydrogenase 155 kDa, albumin 66 kDa, and carboanhydrase 29 kDa; Merck, 69385) were loaded into the Superdex column and an elution spectrum was generated. For each protein marker, the elution volume at the highest absorbance point was taken and plotted against their log_10_MW values. A linear curve was fitted to determine the elution equation for the SEC column, which was then used to predict elution volumes for the FRET (58 kDa) and donor-only proteins (33 kDa).

### Calculation of corrected theoretical mass of proteins

Based on the amino acid sequence, the theoretical mass of the FRET protein was 58767.81 Da. However, since autocatalytic cyclization is required to produce chromophores in the fluorescent proteins, a correction was performed that took into account two mass shifts of 20.77 Da (corresponding to a loss of one oxygen and four to five hydrogen atoms). Therefore, the corrected theoretical mass of the FRET protein after post-translational modifications was determined to be 58726.27 Da. Similarly, the theoretical mass of the donor-only protein was 33277.05 Da. After accounting for one mass shift of 20.77 Da due to post-translational cyclization, the corrected theoretical mass was determined to be 33256.28 Da.

### Calculation of FRET efficiency

FRET efficiency (*E*_*FRET*_) was calculated using the following equation:

$$E_{FRET}=1-\frac{\tau_{DA}}{\tau_{D}}$$

where τ_D_ is the lifetime of the donor-only protein and τ_DA_ is the lifetime of the donor in the presence of the acceptor.

### FRET and donor-only protein conjugation to PEG-8VS

FRET or donor-only protein was treated with 5-fold excess (compared to protein molar concentration) of tris(2-carboxyethyl)phosphine (TCEP, Thermo Fisher, 20490) for 30 min at RT to reduce the cysteine residue at the protein’s C-terminus. TCEP was then removed using desalting columns (Merck, GE17-0851). 20 kDa PEG-8VS (JenKem, ZZ313P138) was dissolved in 30 mM HEPES buffer (pH 8) containing 150 mM NaCl and conjugated to the reduced FRET or donor-only protein at a molar ratio of 12.5:1 (PEG-8VS:protein) at RT by incubating for 1 h. To determine the concentration of conjugates, absorbance was measured at 490 nm (normalized to 30 mM HEPES (pH 8) with 150 mM NaCl) using a UV/Vis spectrophotometer (CLARIOstar). Sample concentration was calculated based on the molar extinction coefficient of SFGFP (45000 M^-1^ cm^-1^) ^[57]^ and the Beer-Lambert law:

$$c=\frac{A}{l\varepsilon }$$

Where *A* is the absorbance of the sample at 490 nm, *l* is the length of the light path (1 cm), *c* is the molar concentration of the sample, and ε is the molar extinction coefficient of the sample.

### FRET sensor and donor-only control purification

Up to 8 arms of PEG-8VS can be functionalized with FRET or donor-only proteins. Thus, protein conjugates have up to eight possible molecular weights, ranging from 78 kDa to 484 kDa. PEG-FRET/donor-only protein conjugates with approximately four unoccupied arms were purified by SEC and used as FRET sensor/donor-only control. SEC was performed at 4 °C at a constant flow at 0.75 mL min^-1^ in 30 mM HEPES (pH 8) with 150 mM NaCl.

### Fluorescence intensity measurements of the FRET sensor

Fluorescence intensity measurements were performed in a solution containing 3.5 μM FRET sensor in 30 mM HEPES buffer supplemented with 150 mM NaCl and 10 mM CaCl_2_ (pH 7.4) with or without 35 nM MMP9 treatment for 1 h. Measurements were performed on CLARIOstar spectrometer with excitation at 462/16 nm and emission scanning between 490 and 650 nm. Fluorescence intensity was normalized to the maximum intensity measurement at 515 nm.

### Fluorescence lifetime measurements (FLIM)

A custom-built time-domain fluorescence lifetime imaging microscope with a time correlated single photon counting (TCSPC) apparatus was used to produce pixel-by-pixel transients. The system is based on a Nikon Ti-E microscope equipped with a X100 1.45NA plan-apo oil objective. A 80 MHz Ti-sapphire laser (Chameleon Vision II, Coherent) was used for excitation, which was detected with a HPM 100-40 hybrid detector (Becker & Hickl). SFGFP excitation was carried out with a low power, two photon 920 nm polarized pulsed laser source, and the subsequent emitted fluorescence passed through a 517 ± 20 nm filter (Semrock) before being detected. A 300 s acquisition time per image was used to ensure adequate photon collection for data analysis while avoiding signal pile-up. All measurements were carried out in chamber slides with a glass bottom (Ibidi, 80827). The temperature was maintained at 37 °C with a thermostatically regulated incubator during imaging.

FLIM data were collected, and processed as described previously. ^[58]^ Data were saved as .ics files and processed using time-resolved imaging (Tri2) software. The pixel fitting method was used, whereby the acquired image was divided into spatial bins, where each bin had a size of 35 x 35 pixels. A minimum of 10,000 photon counts per bin was used to ensure the accuracy of the pixel fitting. To calculate lifetime of each bin, a histogram of photon number (intensity) based on photon arrival times was fitted with a Levenberg-Marquardt (LM) mono-exponential model:

$$f(t)=Z+Ae^{-t/\tau }$$

where *Z* is noise baseline, *t* is time (in ns), *A* is the peak intensity of the exponential, and τ is lifetime (in ns).

The lifetime value of all spatial bins was calculated and is presented as heatmaps. The average lifetime was calculated from all lifetime values presented within the heatmap.

### Assessment of MMP9-mediated FRET protein cleavage

3.5 μM FRET protein or donor-only protein was combined with 35 nM human MMP-9 (Sigma SAE0078) in 30 mM HEPES (pH 7.4) supplemented with 150 mM NaCl and 10 mM CaCl_2_ and incubated at 37 °C for 1 h. The fluorescence lifetime of SFGFP was measured after 15, 30, 45 and 60 min.

### Formation of hydrogels containing the FRET sensor or donor-only control

PEG-peptide conjugates were created, as described previously ^[20, 21]^ by reacting primary amines on lysine residues within either Ac-KDW-ERC-NH2 (non-functional), (RGDSGD)K-GDQGIAGF-ERC-NH2 (adhesive) or Ac-GRDSGK-GPQG↓IWGQ-ERC-NH2 (degradable) peptides (all custom synthesis from Peptide Protein Research, Ltd, >98% purity) with 4-arm 10 kDa PEG nitrophenyl carbonate (PEG-4NPC, JenKem), forming stable carbamate linkages. Purified conjugates (see Table S4) were mixed with FRET-sensors or donor-only controls and 4-arm 20 kDa PEG vinyl sulfone (PEG-4VS, JenKem) in 30 mM HEPES (pH 8) with 1X Hank’s Balanced Salt Solution (HBSS). Total PEG-VS (contributed from the FRET sensor or donor-only control and PEG-4VS) and PEG-peptide conjugates were kept in a stoichiometry ratio of 1:1 and pipetted into glass cylinders (SciQuip, 2090-00608) that had been pre-treated with Sigmacote® (Sigma, SL2), according to the manufacturer’s instructions. After incubation at 37 °C for 1 h, glass cylinders were removed. The FRET sensor or donor-only control containing 2.5% (w/v) hydrogels were then placed in 30 mM HEPES (pH 7.4) with 150 mM NaCl and 10 mM CaCl_2_ for 24 h at 37 °C to allow for hydrogel swelling before fluorescence lifetime of the SFGFP was measured.

### Rheological measurements of hydrogel gelation

Non-degradable hydrogels (Table S4a) with or without the FRET sensor were formed as described above. Hydrogel gelation was assessed on a strain-controlled DHR-3 from TA Instruments using a 8 mm cone with a 0.02-rad angle and plate by carrying out small-amplitude oscillatory time-sweep measurements at a strain of 5% and a constant angular frequency of 1 rad s^−1^. To perform measurements, 90 μL of hydrogel precursor solution was placed in the instrument and paraffin oil was placed around the sample edges to prevent evaporation. Storage modulus G′ and loss modulus G″ were then recorded as a function of time in Trios software (version 5.0.0.44608). Measurements were carried out at 37 °C. Plateau modulus was determined by calculating mean G’ for each hydrogel between 30 and 40 min.

### Fluorescence intensity measurements on hydrogels

Non-degradable hydrogels (Table S4a) containing the FRET sensor were formed as described above. Imaging-based measurements of fluorescence intensity were performed on a Zeiss ApoTome 2 microscope equipped with a 40x objective at 1936 x 1460 pixel resolution. Hydrogels were incubated with or without 35 nM MMP9 in 30 mM HEPES (pH 7.4) with 150 mM NaCl and 10 mM CaCl_2_ for 4 h. Fluorescence was collected through filters specific either for SFGFP (excitation 450-490nm, emission 500-550nm) or mRFP (excitation 559-585 nm, emission 600-690). Fluorescence intensity in each channel was analyzed using Fiji ImageJ, and the acceptor (mRFP)-to-donor (SFGFP) fluorescence intensity ratio was calculated.

### MMP mediated FRET sensor cleavage within the hydrogel

FRET sensor-containing hydrogels with either degradable or non-degradable PEG-peptide conjugates were treated with either 105 nM or 35 nM human MMP9 (Sigma SAE0078) in 30 mM HEPES (pH 7.4) supplemented with 150 mM NaCl and 10 mM CaCl_2_ at 37 °C for up to 240 min. The fluorescence lifetime of SFGFP was measured at regular intervals.

### Cell culture

MMP14 expressing MCF-7 (MCF-MMP14) cells ^[36]^ (a generous gift from Dr Sashko Damjanovski at University of Western Ontario) were cultured in Dulbecco's Modified Eagle Medium (DMEM, Thermo Fisher, 10566016) with 10% fetal bovine serum (FBS, Thermo Fisher, 10270106), 1% antibiotic-antimycotic (ABAM, Thermo Fisher, 15240096) and 1 mg mL^-1^ G418 (VWR, 97064-358). MCF-7 cells were cultured in the same media without G418. Cells were maintained at 37 °C with 5% CO_2_/95% air, and the medium was replaced every 2-3 days.

### Cell encapsulation in FRET sensor-containing hydrogels

1 x 10^6^ cells/mL MCF-MMP14 or MCF-7 cells were mixed with FRET sensors, PEG-peptide conjugates and 20 kDa 4 arm PEG-4VS in 30 mM HEPES (pH 8) with 1X HBSS. Total PEG-VS (contributed from FRET sensor and PEG-4VS) and PEG-peptide conjugates were kept in a stoichiometry ratio of 1:1 and maintained at 37 °C for 1 h to allow hydrogel gelation. The solution was pipetted into glass cylinders (SciQuip, 2090-00608) that had been pre-treated with Sigmacote^®^ (Sigma, SL2), according to the manufacturer’s instructions. After incubation at 37 °C for 1 h, glass cylinders were removed. The resulting FRET sensor-containing hydrogels were cultured in MCF-MMP14 or MCF-7 culture media under standard conditions and fluorescence lifetime measurements carried out after 2, 12 or 24 h.

### Cell viability in hydrogels

1 x 10^6^ cells/mL MCF-MMP14 or MCF-7 cells were encapsulated in hydrogels and cultured in MCF-MMP14 or MCF-7 culture media, as described above. Cell viability was assessed using a LIVE/DEAD Viability/Cytotoxicity Kit (Thermofisher, L3224), according to the manufacturer’s instructions. Hydrogels were washed with phosphate buffered saline (PBS) twice before the addition of ethidium-homodimer (EthD) and calcein-AM (2 μM for both dyes, in phenol red free DMEM, Thermofisher, 21063029). After 30 min at 37 °C, hydrogels were washed twice with PBS and placed in phenol red free DMEM media. Fluorescence images were taken on a Zeiss apotome microscope.

### FLIM image capture and analysis of cell-laden hydrogels

Individual cells were first identified under bright field conditions and then brought into focus by maximizing the cell edge contrast. The detection path was then switched to acquire images using the TCSPC detectors. Imaging data was acquired by rapidly scanning the excitation across the field of view. FLIM images were captured in 20 μm x 20 μm areas around cells as described above, using a 300 s acquisition time.

FLIM images at the cell-hydrogel interface were first processed using a top-hat filtering strategy to enhance the signal-to-background ratio, enabling edge detection using the Canny edge detection method. ^[39]^ After identifying the cell edge, the primary contour was drawn. Subsequent contours were then drawn by constructing a unit normal to the tangent of the primary contour at each pixel, thus creating the next contour equidistant at all points from the initial contour. This process was repeated forming a continuum of contours radiating outward form the primary contour. Given that the size of the laser spot was the same as the width of four contours, pixel binning was performed, which summed every four consecutive contours, avoiding data overinterpretation. This approach provided sufficiently high spatial resolution orthogonal to the cell edge and an adequate number of photons to produce robust fittings.

### Statistical analyses

Data are presented as means ± standard deviations, unless noted otherwise. Statistical analyses were carried out using GraphPad Prism software (v 10.1.0). Comparisons between two groups were performed using an unpaired two-tailed t-test. Comparisons were considered significant if *p* < 0.05.

## Supplementary Material

Supplementary code

Supplementary code

Supplementary Information

## Data Availability

The data that support the findings of this study are available from the corresponding author upon reasonable request. The MATLAB code used to analyze the FLIM datasets is available within the Supplementary Information and at https://github.com/eileengentleman/FLIM-code

## References

[1] Henderson NC, Rieder F, Wynn TA (2020). Nature.

[2] Gross J, Lapiere CM (1962). Proc Natl Acad Sci U S A.

[3] Blache U, Stevens MM, Gentleman E (2020). Nat Biomed Eng.

[4] Rabkin SW (2017). Prog Mol Biol Transl Sci.

[5] Elkington PT, Friedland JS (2006). Thorax.

[6] Kessenbrock K, Plaks V, Werb Z (2010). Cell.

[7] Page-McCaw A, Ewald AJ, Werb Z (2007). Nat Rev Mol Cell Biol.

[8] Chakraborti S, Mandal M, Das S, Mandal A, Chakraborti T (2003). Mol Cell Biochem.

[9] Brett CMA, Brett AMO (2007). Encyclopedia of Electrochemistry.

[10] Homola J (2008). Chem Rev.

[11] Mohseni S, Moghadam TT, Dabirmanesh B, Jabbari S, Khajeh K (2016). Biosens Bioelectron.

[12] Fonovic M, Bogyo M (2008). Expert Rev Proteomics.

[13] Knapinska A, Fields GB (2012). Chembiochem.

[14] Jares-Erijman EA, Jovin TM (2003). Nat Biotechnol.

[15] Nedbal J, Visitkul V, Ortiz-Zapater E, Weitsman G, Chana P, Matthews DR, Ng T, Ameer-Beg SM (2015). Cytometry A.

[16] Pelet S, Previte MJ, So PT (2006). J Biomed Opt.

[17] Law AL, Jalal S, Pallett T, Mosis F, Guni A, Brayford S, Yolland L, Marcotti S, Levitt JA, Poland SP, Rowe-Sampson M (2021). Nat Commun.

[18] Chandler J, Treacy C, Ameer-Beg S, Ehler E, Irving M, Kampourakis T (2023). Proc Natl Acad Sci U S A.

[19] Lutolf MP, Hubbell JA (2003). Biomacromolecules.

[20] Jowett GM, Norman MDA, Yu TTL, Rosell Arevalo P, Hoogland D, Lust ST, Read E, Hamrud E, Walters NJ, Niazi U, Chung MWH (2021). Nat Mater.

[21] Lust ST, Hoogland D, Norman MDA, Kerins C, Omar J, Jowett GM, Yu TTL, Yan Z, Xu JZ, Marciano D, da Silva RMP (2021). ACS Biomater Sci Eng.

[22] Foyt DA, Norman MDA, Yu TTL, Gentleman E (2018). Adv Healthc Mater.

[23] Blache U, Ford EM, Ha B, Rijns L, Chaudhuri O, Dankers PYW, Kloxin AM, Snedeker JG, Gentleman E (2022). Nat Rev Methods Primers.

[24] Patterson J, Hubbell JA (2010). Biomaterials.

[25] Campbell RE, Tour O, Palmer AE, Steinbach PA, Baird GS, Zacharias DA, Tsien RY (2002). Proc Natl Acad Sci U S A.

[26] Hunt J, Keeble AH, Dale RE, Corbett MK, Beavil RL, Levitt J, Swann MJ, Suhling K, AmeerBeg S, Sutton BJ, Beavil AJ (2012). J Biol Chem.

[27] Datta R, Heaster TM, Sharick JT, Gillette AA, Skala MC (2020). J Biomed Opt.

[28] Lakowicz JR (2006). Principles of Fluorescence Spectroscopy.

[29] Chen H, Puhl HL, Koushik SV, Vogel SS, Ikeda SR (2006). Biophys J.

[30] Blackford SJI, Yu TTL, Norman MDA, Syanda AM, Manolakakis M, Lachowski D, Yan Z, Guo Y, Garitta E, Riccio F, Jowett GM (2023). Biomaterials.

[31] Lutolf MP, Lauer-Fields JL, Schmoekel HG, Metters AT, Weber FE, Fields GB, Hubbell JA (2003). Proc Natl Acad Sci U S A.

[32] Ferreira SA, Motwani MS, Faull PA, Seymour AJ, Yu TTL, Enayati M, Taheem DK, Salzlechner C, Haghighi T, Kania EM, Oommen OP (2018). Nat Commun.

[33] Cruz-Acuna R, Quiros M, Farkas AE, Dedhia PH, Huang S, Siuda D, Garcia-Hernandez V, Miller AJ, Spence JR, Nusrat A, Garcia AJ (2017). Nat Cell Biol.

[34] Figueira RC, Gomes LR, Neto JS, Silva FC, Silva ID, Sogayar MC (2009). BMC Cancer.

[35] Kohrmann A, Kammerer U, Kapp M, Dietl J, Anacker J (2009). BMC Cancer.

[36] Cepeda MA, Pelling JJ, Evered CL, Williams KC, Freedman Z, Stan I, Willson JA, Leong HS, Damjanovski S (2016). Mol Cancer.

[37] Schultz KM, Kyburz KA, Anseth KS (2015). Proc Natl Acad Sci U S A.

[38] Long H, Vos BE, Betz T, Baker BM, Trappmann B (2022). Adv Sci (Weinh).

[39] Canny J (1986). IEEE Trans Pattern Anal Mach Intell.

[40] Shin DS, Tokuda EY, Leight JL, Miksch CE, Brown TE, Anseth KS (2018). ACS Biomater Sci Eng.

[41] Murphy G, Nagase H (2011). FEBS J.

[42] Sander EA, Nauman EA (2003). Crit Rev Biomed Eng.

[43] Rehmann MS, Skeens KM, Kharkar PM, Ford EM, Maverakis E, Lee KH, Kloxin AM (2017). Biomacromolecules.

[44] Mazzoccoli JP, Feke DL, Baskaran H, Pintauro PN (2010). J Biomed Mater Res A.

[45] Baugh MD, Perry MJ, Hollander AP, Davies DR, Cross SS, Lobo AJ, Taylor CJ, Evans GS (1999). Gastroenterology.

[46] Shimshoni E, Yablecovitch D, Baram L, Dotan I, Sagi I (2015). Gut.

[47] Fata JE, Werb Z, Bissell MJ (2004). Breast Cancer Res.

[48] Blache U, Vallmajo-Martin Q, Horton ER, Guerrero J, Djonov V, Scherberich A, Erler JT, Martin I, Snedeker JG, Milleret V, Ehrbar M (2018). EMBO Rep.

[49] Ferreira SA, Faull PA, Seymour AJ, Yu TTL, Loaiza S, Auner HW, Snijders AP, Gentleman E (2018). Biomaterials.

[50] Packard BZ, Artym VV, Komoriya A, Yamada KM (2009). Matrix Biol.

[51] Ouyang M, Lu S, Li XY, Xu J, Seong J, Giepmans BN, Shyy JY, Weiss SJ, Wang Y (2008). J Biol Chem.

[52] Ouyang M, Sun J, Chien S, Wang Y (2008). Proc Natl Acad Sci U S A.

[53] Lu S, Wang Y, Huang H, Pan Y, Chaney EJ, Boppart SA, Ozer H, Strongin AY, Wang Y (2013). PLoS One.

[54] Hodgson L, Pertz O, Hahn KM (2008). Methods Cell Biol.

[55] Leonard J, Dumas N, Causse JP, Maillot S, Giannakopoulou N, Barre S, Uhring W (2014). Lab Chip.

[56] Studier FW (2014). Methods Mol Biol.

[57] Suzuki T, Arai S, Takeuchi M, Sakurai C, Ebana H, Higashi T, Hashimoto H, Hatsuzawa K, Wada I (2012). PLoS One.

[58] Barber PR, Ameer-Beg SM, Gilbey J, Carlin LM, Ng TC, Vojnovic B (2009). J R Soc Interface.

