# Supplementary material for: FRET Sensor-Modified Synthetic Hydrogels for Real-Time Monitoring of Cell-Derived Matrix Metalloproteinase Activity using Fluorescence Lifetime Imaging
Source: Adv Funct Mater. Author manuscript; Available in PMC 2024 May 22. (PMC7615971; doi:10.1002/adfm.202309711)
Supplement: Supplementary code [file EMS194565-supplement-Supplementary_code.docx]

function [] = FLIM_kymo_analysis(foldername,orient,denoise)

%FLIM_Kymo_analysis % Script designed to generate a kymograph from Flim data

% Script designed to take the tiffs in the current folder, load them in and

% allow the user to define a region from which a kymogrpah can be made.

% Data is then saved to be loaded into FLIMJ.

% NB the initial tiffs are generated by taking the SDT file and opening it

% in FIJI (bio-formats plugin)

% Inputs:

% fname = filename to analyse (including .sdt)

% orient = allows the direction of contours to be reversed (1 = normal op 0 = inverted)

% denoise = turn on (1) or off(0) denoising

% copyright (c) 2023 Thomas Kavanagh

% All rights reserved.

% This source code is licensed under the CC-BY-SA-4.0 license found in the

% LICENSE file in the root directory of this source tree.

% Input check

if ~exist('foldername', 'var') || ~ischar(foldername) || ~isfolder(foldername) || strcmp(foldername,'foldername')

foldername = uigetdir(pwd,'Select folder containing images');

if foldername==0

disp('User aborted')

return

end

cd(foldername)

f_no = length(dir('*.tif'));

if f_no<1

disp('No tifs in folder, rerun program')

return

else

addpath(genpath(foldername))

end

else

end

if ~exist('orient', 'var')

orient = 1;

else

end

if ~exist('denoise', 'var')

denoise = 0;

else

end

% Initial clears

close all

save_fig = 0;

dual_cont = 0;

% User set Variables

cont_num = 160; % number of contour (NB contours are wide)

sum_reg_no=4; % number of consectutive contours to sum over (NB to have thicker contours set this higher)

int_cont_no = 12;

% Variable definitions

cont_gaps = 1; % gaps between contours. Always set to = 1

im_data = zeros(255,256,256);

sum_im = double(zeros(255,256));

corr = 0;

thresh_factor = 2;

% Tophat filter element

se_dsk_hat = strel('disk',2,0);

% Contour fill element

se_dsk_cont = strel('disk',cont_gaps+5,0);

% smoothing coeff (Savitzky-Golay)

windowWidth = 21;

polynomialOrder = 3;

% OTF filtering

Lx = 256;

Ly = 255;

px = floor(-Lx/2)+1:1:floor(Lx/2);

qx = floor(-Ly/2)+1:1:floor(Ly/2);

kx = px/(Lx*83e-9);

ky = (qx/(Ly*83e-9))';

kr = sqrt((kx.^2)+(ky.^2));

T = 900e-9/5.6*1.49;

otf_mask = 0.5 + 0.5*(cos((pi*T)*kr));

% Setup parameter file

param = zeros(5,1); % to record the parameters used --> feeds into results file

param(1,1) = cont_num;

param(2,1) = int_cont_no;

param(3,1) = sum_reg_no;

% wavelet decomp param

sorh = 's'; % soft threhold

wname = 'sym2'; % symlet 2

level=1; % dempose into 2 comp noise and other ( i.e. course decomp)

%% Load data

ims = imageDatastore(foldername); % creating data store from foldername

t_fr = numel(ims.Files);

for l = 1:t_fr

[im, info] = readimage(ims,l);

im = double(im(1:255,:));

fr_no = info.Filename;

fr_no = str2double(fr_no(end-7:end-4));

im_data(:,:,fr_no+1) = im;

end

[y_sz, x_sz, nrBins] = size(im_data);

im_data_deN = zeros(size(im_data));

% No. of frame

if denoise ==1

for i = 1:nrBins

im_data_deN(:,:,i) = flim_denoise(im_data(:,:,i),3,1);

end

else

end

sum_im = sum(im_data,3);

sum_im = sum_im(1:255,1:256);

Lx = size(sum_im,2);

Ly = size(sum_im,1);

sum_im_inv = max(max(sum_im))./sum_im;

sum_im_inv(sum_im_inv==inf)= max(max(sum_im));

sum_im_inv_filt = mat2gray(sum_im_inv);

sum_im_inv_filt = imtophat(sum_im_inv_filt,se_dsk_hat);

mask_freq_im = fftshift(fft2(sum_im_inv_filt));

mask_freq_im = mask_freq_im.*otf_mask;

sum_im_inv_filt = abs(ifft2(fftshift(mask_freq_im)));

[C,S] = wavedec2(sum_im_inv_filt,level,wname);

thr = wthrmngr('dw2ddenoLVL','penalhi',C,S,3);

[sum_im_inv_filt,~,~] = wdencmp('lvd',C,S,wname,level,thr,sorh);

while corr == 0

[im_thresh,prim_cont,edge_cont] = mask_and_cont(sum_im_inv_filt,thresh_factor);

[obs,obs_num] = bwlabel(im_thresh,8);

figure(2)

subplot(1,2,1)

imagesc(sum_im_inv);title('Inverted sum image')

hold ON

plot(prim_cont(:,2),prim_cont(:,1),'k','LineWidth',2);

axis image

subplot(1,2,2)

imagesc(im_thresh);title('mask image')

axis image

set(gcf, 'Units', 'Normalized', 'OuterPosition', [0, 0.08, 1, 0.92]);

pause(0.4)

correct = questdlg('Increase or decrease cell mask threshold','Correct?','Inrease', 'Decrease','Correct', 'Decrease');

switch correct

case 'Inrease'

disp([correct ' Threshold increased'])

corr = 0;

thresh_factor = thresh_factor+0.1;

case 'Decrease'

disp([correct ' Threshold decreased'])

corr = 0;

thresh_factor = thresh_factor-0.1;

case'Correct'

disp([correct ' User verified mask is correct'])

corr = 1;

[obs,obs_num] = bwlabel(im_thresh,8);

end

end

dual = questdlg('Include internal contours or just external','Dual contours?','Extenal only', 'Both', 'Extenal only');

switch dual

case 'Extenal only'

disp('External contours only')

dual_cont =0;

param(2,1) = 0;

total_cont_no = cont_num;

case 'Both'

disp('Both external and internal contours')

dual_cont = 1;

total_cont_no = cont_num + int_cont_no;

end

if obs_num == 1

pause(1.0)

close all

[N,Vertices,Lines] = cont_extrapolate(prim_cont,im_thresh,cont_gaps); % See script for comments

if orient ==0

N = -N;

else

end

if dual_cont == 0

new_im_thresh = im_thresh;

new_pos = {cont_num,2};

for ii = 1:cont_num

new_cont = [Vertices(:,1)+ii*cont_gaps*-N(:,1), Vertices(:,2)+ii*cont_gaps*-N(:,2)];

smoothY = sgolayfilt(new_cont(:,2), polynomialOrder, windowWidth);

smoothX = sgolayfilt(new_cont(:,1), polynomialOrder, windowWidth);

new_cont_rd = [smoothX smoothY];

new_cont_rd = round(new_cont_rd);

new_cont_rd(new_cont_rd<1)=1;

new_cont_rd(new_cont_rd>255)=255;

cont_im = full(sparse(new_cont_rd(:,2), new_cont_rd(:,1),true,Ly,Lx));

new_im_thresh = logical(cont_im + new_im_thresh); % has to be the previous iteration;

new_im_thresh = imerode(imfill(imdilate(new_im_thresh, se_dsk_cont),'holes'), se_dsk_cont);

[contour,edge_contour] = cont_retrieval(new_im_thresh);

new_pos(ii,1) = {contour(:,1)};

new_pos(ii,2) = {contour(:,2)};

end

else

new_im_thresh = im_thresh;

new_pos = {total_cont_no,2};

for ii = 1:total_cont_no

neg_index = ii-int_cont_no;

new_cont = [Vertices(:,1)+neg_index*cont_gaps*N(:,1), Vertices(:,2)+neg_index*cont_gaps*N(:,2)]; % NB could employ boundary to remove 'coils' in the boundary but it may alter the data too much

smoothY = sgolayfilt(new_cont(:,2), polynomialOrder, windowWidth);

smoothX = sgolayfilt(new_cont(:,1), polynomialOrder, windowWidth);

new_cont_rd = [smoothX smoothY];

new_cont_rd = round(new_cont_rd);

new_cont_rd(new_cont_rd<1)=1;

new_cont_rd(new_cont_rd>255)=255;

cont_im = full(sparse(new_cont_rd(:,2), new_cont_rd(:,1),true,Ly,Lx));

new_im_thresh = logical(cont_im + new_im_thresh); % has to be the previous iteration;

new_im_thresh = imerode(imfill(imdilate(new_im_thresh, se_dsk_cont),'holes'), se_dsk_cont);

[contour,edge_contour] = cont_retrieval(new_im_thresh);

new_pos(ii,1) = {contour(:,1)};

new_pos(ii,2) = {contour(:,2)};

end

end

elseif obs_num == 2

edge_cont = [];

im_thresh_1 = obs; im_thresh_1(im_thresh_1~=1) = 0;

im_thresh_1 = logical(im_thresh_1);

im_thresh_2 = obs; im_thresh_2(im_thresh_2~=2) = 0;

im_thresh_2 = logical(im_thresh_2);

edge_cont(1,:,:) = edge(im_thresh_1);

edge_cont(2,:,:) = edge(im_thresh_2);

figure(2)

subplot(1,3,1)

imagesc(sum_im_inv);title('Inverted sum image')

axis image

subplot(1,3,2)

imagesc(squeeze(edge_cont(1,:,:)))

axis image

subplot(1,3,3)

imagesc(squeeze(edge_cont(2,:,:)))

axis image

comb_conts = [];

for j = 1:2

[prim_cont,edge_contour] = cont_retrieval(edge_cont(j,:,:));

[N,Vertices,Lines] = cont_extrapolate(prim_cont,im_thresh,cont_gaps); % Could iterate this to gen the diff cont

if orient ==0

N = -N;

else

end

if dual_cont == 0

new_im_thresh = im_thresh;

new_pos = {cont_num,2};

for ii = 1:cont_num

new_cont = [Vertices(:,1)+ii*cont_gaps*N(:,1), Vertices(:,2)+ii*cont_gaps*N(:,2)];

smoothY = sgolayfilt(new_cont(:,2), polynomialOrder, windowWidth);

smoothX = sgolayfilt(new_cont(:,1), polynomialOrder, windowWidth);

new_cont_rd = [smoothX smoothY];

new_cont_rd = round(new_cont_rd);

new_cont_rd(new_cont_rd<1)=1;

new_cont_rd(new_cont_rd>256)=256;

cont_im = full(sparse(new_cont_rd(:,2), new_cont_rd(:,1),true,Ly,Lx));

new_im_thresh = logical(cont_im + new_im_thresh); % has to be the previous iteration;

new_im_thresh = imerode(imfill(imdilate(new_im_thresh, se_dsk_cont),'holes'), se_dsk_cont);

[contour,edge_contour] = cont_retrieval(new_im_thresh);

new_pos(ii,1) = {contour(:,1)};

new_pos(ii,2) = {contour(:,2)};

end

else

new_pos = {total_cont_no,2};

new_im_thresh = im_thresh;

for ii = 1:total_cont_no

neg_index = ii-int_cont_no;

new_cont = [Vertices(:,1)+neg_index*cont_gaps*N(:,1), Vertices(:,2)+neg_index*cont_gaps*N(:,2)]; % NB could employ boundary to remove 'coils' in the boundary but it may alter the data too much

smoothY = sgolayfilt(new_cont(:,2), polynomialOrder, windowWidth);

smoothX = sgolayfilt(new_cont(:,1), polynomialOrder, windowWidth);

new_cont_rd = [smoothX smoothY];

new_cont_rd = round(new_cont_rd);

new_cont_rd(new_cont_rd<1)=1;

new_cont_rd(new_cont_rd>256)=256;

cont_im = full(sparse(new_cont_rd(:,2), new_cont_rd(:,1),true,Ly,Lx));

new_im_thresh = logical(cont_im + new_im_thresh); % has to be the previous iteration;

new_im_thresh = imerode(imfill(imdilate(new_im_thresh, se_dsk_cont),'holes'), se_dsk_cont);

[contour,edge_contour] = cont_retrieval(new_im_thresh);

new_pos(ii,1) = {contour(:,1)};

new_pos(ii,2) = {contour(:,2)};

end

end

comb_conts = [comb_conts; new_pos];

end

new_pos = comb_conts; clear comb_conts;

edge_cont = squeeze(edge_cont(1,:,:))+ squeeze(edge_cont(2,:,:));

total_cont_no = length (new_pos);

else

disp('Image is either empty or contains to many cells to process')

end

figure(3)

subplot(1,2,1)

imagesc(sum_im_inv)

axis image

colormap(parula)

colorbar

title('Sum image')

subplot(1,2,2)

imagesc(edge_cont)

axis image

set(gcf, 'Units', 'Normalized', 'OuterPosition', [0, 0.08, 1, 0.92]);

title('Raw Primary contour')

figure(6)

subplot(1,2,1)

imagesc(sum_im_inv)

hold ON

plot(prim_cont(:,2),prim_cont(:,1),'w','LineWidth',2);

axis image

xlim([0 256]);

ylim([0 256]);

set(gca,'YDir','reverse')

set(gcf, 'Units', 'Normalized', 'OuterPosition', [0, 0.08, 1, 0.92]);

subplot(1,2,2)

imagesc(sum_im_inv)

colormap(parula)

figure(4)

subplot(1,2,2)

imagesc(sum_im_inv)

hold ON

for kk = 1:total_cont_no

figure(4)

subplot(1,2,1)

hold ON

plot(new_pos{kk,2},new_pos{kk,1},'k','LineWidth',2);

axis image

xlim([0 256]);

ylim([0 256]);

set(gca,'YDir','reverse')

subplot(1,2,2)

plot(new_pos{kk,2},new_pos{kk,1},'k','LineWidth',2);

hold ON

axis image

xlim([0 256]);

ylim([0 256]);

set(gca,'YDir','reverse')

set(gcf, 'Units', 'Normalized', 'OuterPosition', [0, 0.08, 1, 0.92]);

figure(6)

hold ON

plot(new_pos{kk,2},new_pos{kk,1},'w','LineWidth',2);

axis image

xlim([0 256]);

ylim([0 256]);

set(gca,'YDir','reverse')

title('All contours overlaid on sum image')

end

% pause ()

save('cont_pos.mat', 'new_pos')

fl_save = questdlg('Write kymo files, save figures and parameters?','Save?','Yes','No','No');

switch fl_save

case 'Yes'

disp([fl_save ' Results saved'])

save_fig = 1;

case 'No'

disp([fl_save ' Results not saved'])

save_fig = 0;

end

if save_fig == 1

mkdir('kymo images');

cd ('kymo images');

for l = 1:nrBins % saving data into another folder that is then fd into FLIMj

if denoise ==1

im = im_data_deN(:,:,l);

im_kymo_bin = zeros(1,total_cont_no);

for jj = 1:total_cont_no

ind = sub2ind(size(im),new_pos{jj,2},new_pos{jj,1});

im_kymo_bin(1,jj) = sum(im(ind),'native');

end

im_kymo_bin = single(im_kymo_bin);

t = Tiff([num2str(l),'.tif'], 'w');

tagstruct.ImageLength = 1;

tagstruct.ImageWidth = total_cont_no;

tagstruct.Compression = Tiff.Compression.None;

tagstruct.SampleFormat = Tiff.SampleFormat.IEEEFP;

tagstruct.Photometric = Tiff.Photometric.MinIsBlack;

tagstruct.BitsPerSample = 32;

tagstruct.SamplesPerPixel = 1;

tagstruct.PlanarConfiguration = Tiff.PlanarConfiguration.Chunky;

tagstruct.MinSampleValue = 0.0;

tagstruct.MaxSampleValue = 50000.0;

t.setTag(tagstruct);

t.write(im_kymo_bin);

t.close();

else

im = uint16(im_data(:,:,l));

im_kymo_bin = uint16(zeros(1,total_cont_no));

for jj = 1:total_cont_no

ind = sub2ind(size(im),new_pos{jj,1},new_pos{jj,2});

im_kymo_bin(1,jj) = sum(im(ind),'native');

end

im_kymo_bin = reshape(im_kymo_bin,sum_reg_no,(total_cont_no/sum_reg_no));

im_kymo_bin = sum(im_kymo_bin,1,'native');

imwrite(im_kymo_bin,[num2str(l),'.tif']);

end

end

cd ../

param(4,1) = thresh_factor;

param(5,1) = 12;

T = table (param, 'RowNames', {'cont_num';'int_cont_no';'sum_reg_no';'thresh_factor';'Version_no'});

writetable(T,'param_file.txt','Delimiter','tab','WriteRowNames', true)

savefig(3,'Sum and raw primary contour.fig')

savefig(6,'Smoothed primary contour and all contours overlaid.fig')

saveas(3,'Sum and raw primary contour.tif')

saveas(6,'Smoothed primary contour and all contours overlaid.tif')

cd ../

else

end

end

function [contour,edge_contour] = cont_retrieval(thresholded_image)

% Sub-function used to retrieve the contour given an binary image

edge_contour = edge(squeeze(thresholded_image));

[yC, xC] = find(edge_contour);

contour = [yC xC];

contour = sortrows(contour,1);

numPoints = length(contour);

dummy_im = false(size(edge_contour,1)-2,size(edge_contour,2)-2);

dummy_im = padarray(dummy_im, ones(1,2), 1, 'both');

[K,L] = find((edge_contour.*dummy_im));

if ~isempty(K)

edge_pts = sortrows([K L],1);

start_pt = find(contour(:,1)== edge_pts(1,1) & contour(:,2)== edge_pts(1,2));

contour = circshift(contour,(1+numPoints-start_pt));

else

end

vis_ord = cont_search(contour(:,2),contour(:,1),numPoints); % travelling salesman

vis_ord = fliplr(vis_ord);

contour = contour(vis_ord,:);

end

function [Normals,Vertices,Lines] = cont_extrapolate(primary_contour,thresholded_image, contour_width)

%Produces the normals for the input primary contour

windowWidth = 21;

polynomialOrder = 2;

smoothX = sgolayfilt(primary_contour(:,2), polynomialOrder, windowWidth);

smoothY = sgolayfilt(primary_contour(:,1), polynomialOrder, windowWidth);

Vertices=[smoothX, smoothY];

Lines=[(1:size(Vertices,1))' (2:size(Vertices,1)+1)'];

Lines(end,2)=1;

Normals=LineNormals2D(Vertices,Lines);

[im_x,im_y] =find(thresholded_image);

shape = alphaShape(im_y,im_x);

pos_check = [Vertices(1,1)+contour_width*Normals(1,1), Vertices(1,2)+contour_width*Normals(1,2)];

if inShape(shape,pos_check(1,1),pos_check(1,2))

Normals = Normals*(-1);

else

end

end

function [thresholded_image,primary_contour,edge_contour] = mask_and_cont(sum_im_inv_filt,threshold_factor)

%Threshold an image, located the edge of an object and produce an outline

%% Erode and dil morph elements

se_sub90 = strel('line', 10, 90);

se0_sub = strel('line',10, 0);

seD_sub = strel('diamond',10);

se_dsk = strel('disk',1,0);

%%

[~, threshold] = edge(sum_im_inv_filt, 'canny'); % Standard Canny edge dectection approach

thresholded_image = edge(sum_im_inv_filt,'canny',threshold_factor*threshold,0.2);

thresholded_image = imdilate(thresholded_image, se_dsk);

thresholded_image = bwareaopen(thresholded_image ,60);

thresholded_image = imfill(thresholded_image, 'holes');

thresholded_image = imdilate(thresholded_image, [se_sub90 se0_sub]);

thresholded_image = imerode(thresholded_image,seD_sub);

thresholded_image = bwareaopen(thresholded_image ,800);

[primary_contour,edge_contour] = cont_retrieval(thresholded_image);

end

% copyright (c) 2023 Thomas Kavanagh
