# Supplementary material for: FRET Sensor-Modified Synthetic Hydrogels for Real-Time Monitoring of Cell-Derived Matrix Metalloproteinase Activity using Fluorescence Lifetime Imaging
Source: Adv Funct Mater. Author manuscript; Available in PMC 2024 May 22. (PMC7615971; doi:10.1002/adfm.202309711)
Supplement: Supplementary Information [file EMS194565-supplement-Supplementary_Information.pdf]

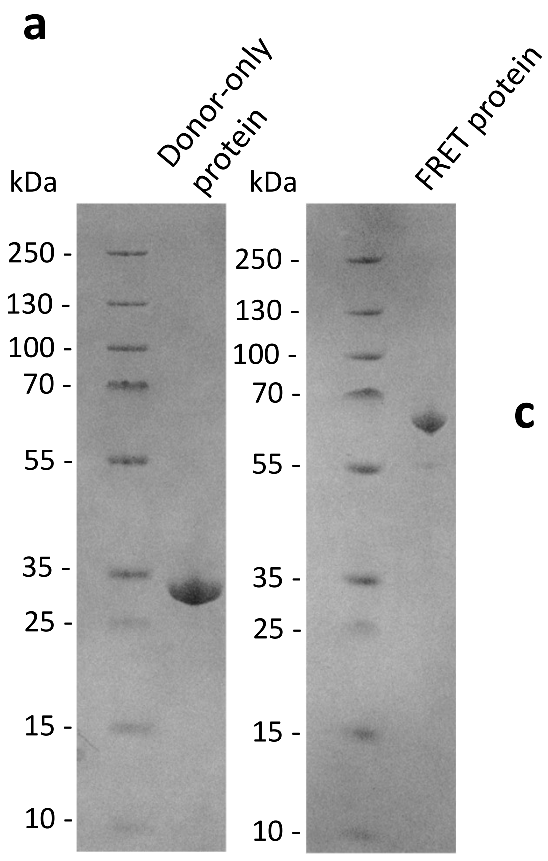

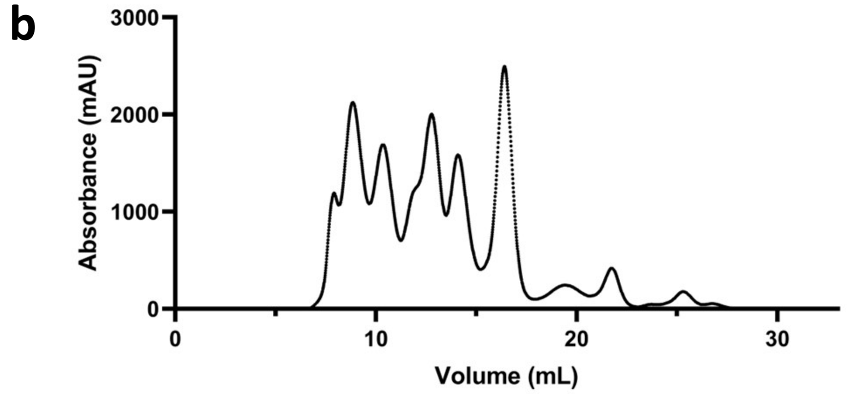

c

	MW of standard marker (kDa)	$\log_{10}$MW of standard marker	Elution volume (mL)
Standard marker 1	669	2.82	8.85
Standard marker 2	443	2.64	10.35
Standard marker 3	150	2.17	12.77
Standard marker 4	66	1.82	14.09
Standard marker 5	29	1.46	16.40

d

	MW of protein (kDa)	Theoretical elution volume (mL)
**Donor-only protein**	33	16.03
**FRET protein**	58	14.73

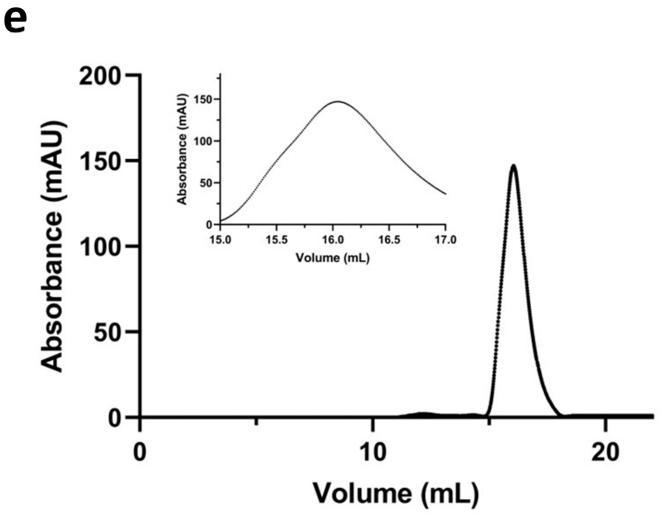

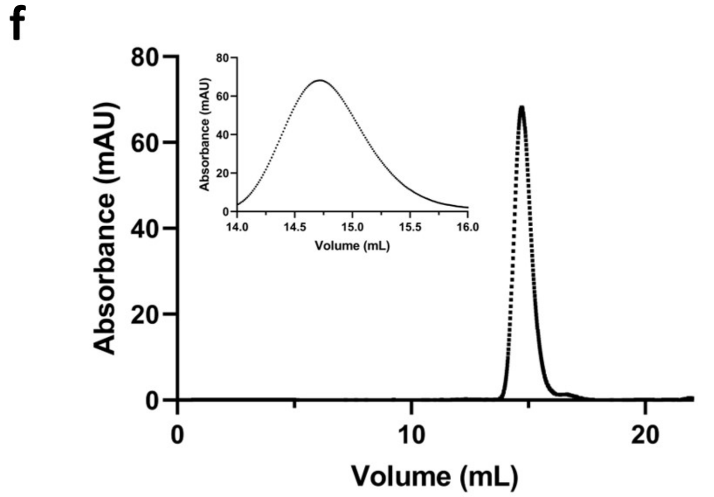

Supplementary Fig. 1: Confirmation of synthesis of FRET protein. **a**. SDS-PAGE of donor-only protein (SFGFP) showing a band at 33 kDa, and FRET protein (RFP-peptide-SFGFP) showing a band at 58 kDa. **b**. SEC elution spectra of five standard protein markers. **c**. Table detailing standard marker MW and corresponding elution volumes, which were used to calculate the theoretical elution volume of the donor-only and FRET proteins based on their MW. **d**. MW and theoretical elution volume of donor-only and FRET proteins. **e**. Elution spectra of the donor-only protein with inset highlighting that the product was eluted at 16.04 mL, as predicted by its MW. **f**. Elution spectra of the FRET protein as shown in Fig. 1d with inset highlighting that the product was eluted at 14.71 mL, as predicted by its MW.

a

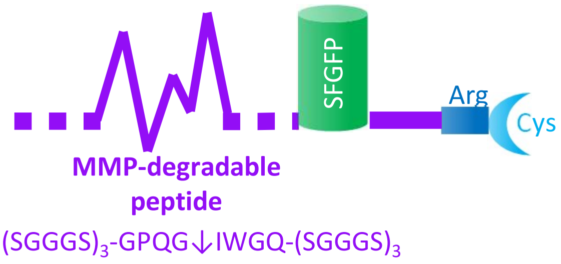

b

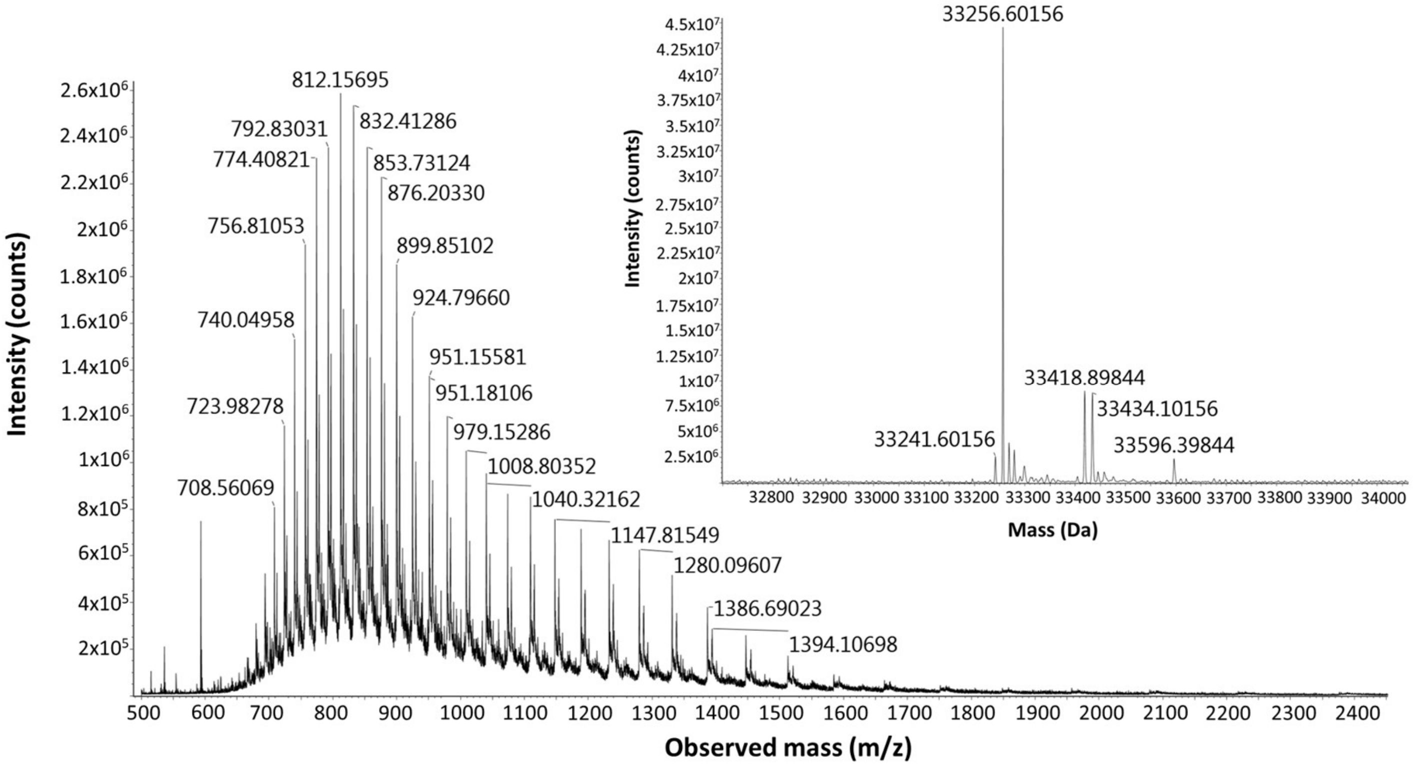

Supplementary Figure 2:. **a**. Design of the donor-only protein, and **b**. Mass spectrometry spectrum of the donor-only protein, with inset showing the deconvoluted spectrum. The theoretical mass of the protein based on its amino acid sequence and after cyclization correction was 33256.28 Da. This matched that of the deconvoluted mass identified in the spectrum (33256.60 Da).

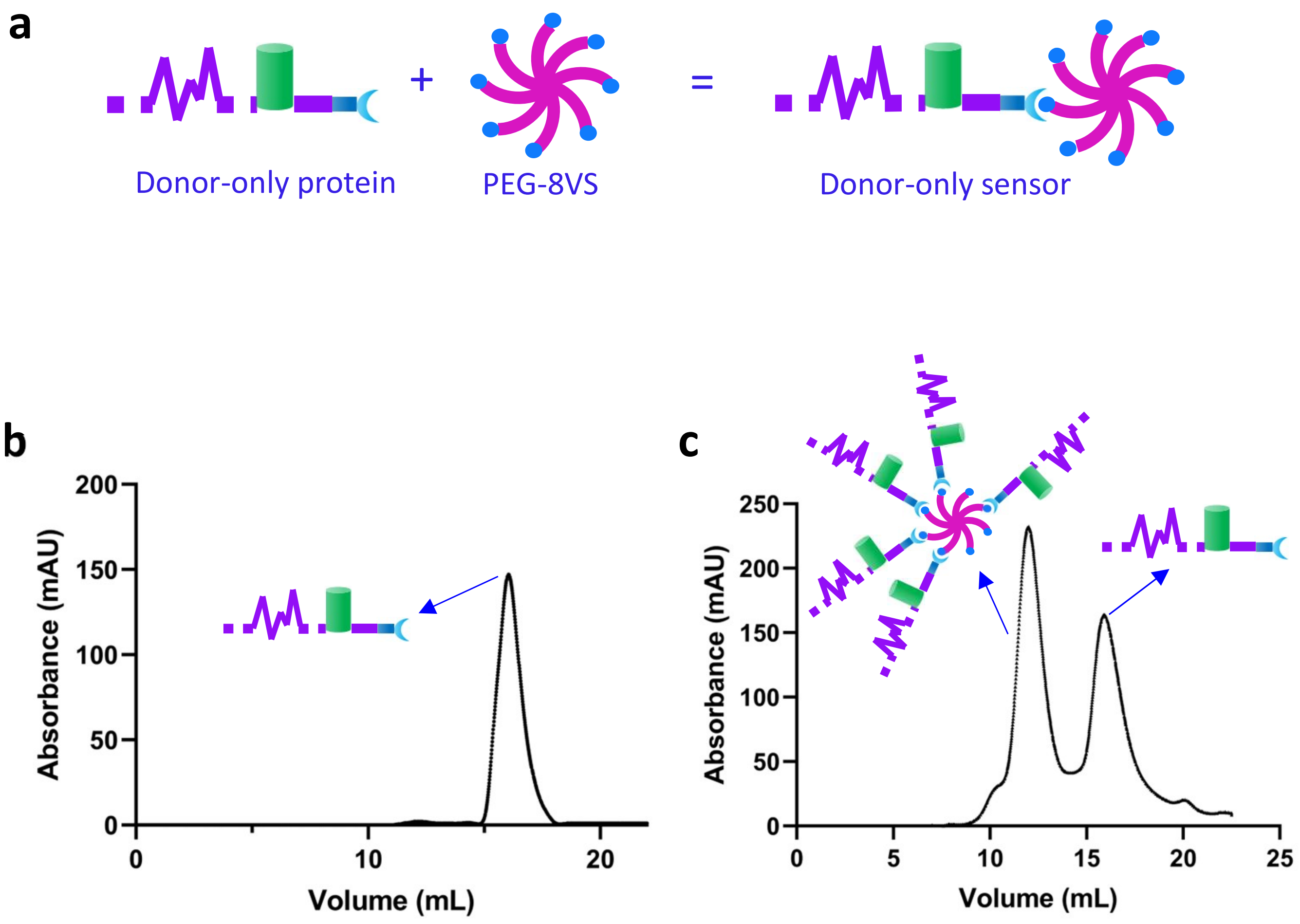

Supplementary Figure 3: **a.** Schematic showing the conjugation of the donor-only protein via a C-terminal cysteine to vinyl sulfone (VS) to create the donor-only sensor. **b**. SEC elution spectra of donor-only protein showing the highest absorbance at an elution volume of 16.04 mL (as in Supplementary Fig. 1e). SEC elution spectra of donor-only protein and PEG-8VS conjugation mixture after 1 hour incubation. The peak at an elution volume of 11.99 mL corresponds to a PEG molecule with 5 arms conjugated to the donor-only protein. The peak at an elution volume of 15.91 mL corresponds to unreacted donor-only protein.

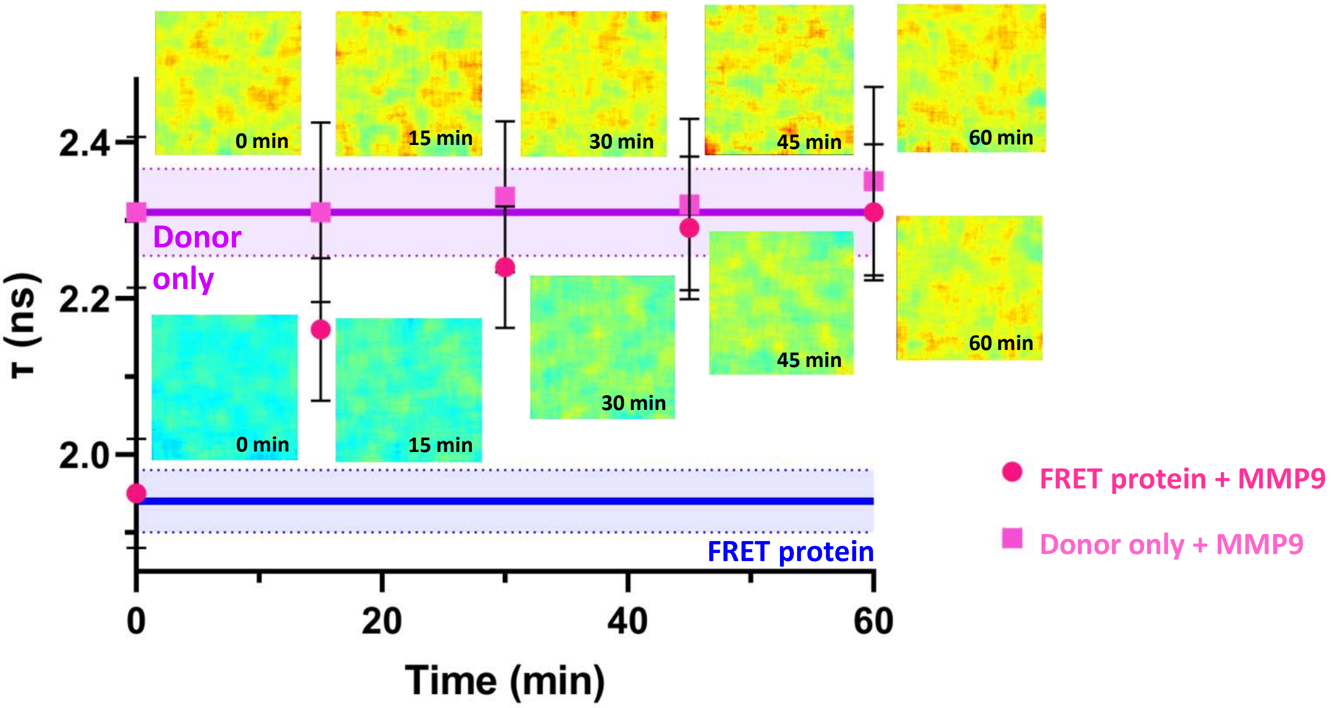

Supplementary Fig. 4: Donor lifetime, τ (mean ± S.D.) of the FRET protein and donor-only protein control treated with 35nM MMP9 measured over 60 minutes (as shown in Fig. 2c), overlaid with example heat maps. The lifetime of the FRET protein and donor-only controls in the absence of MMP9 treatment are shown for comparison (mean ± S.D., shaded area). n=3, for all groups.

a

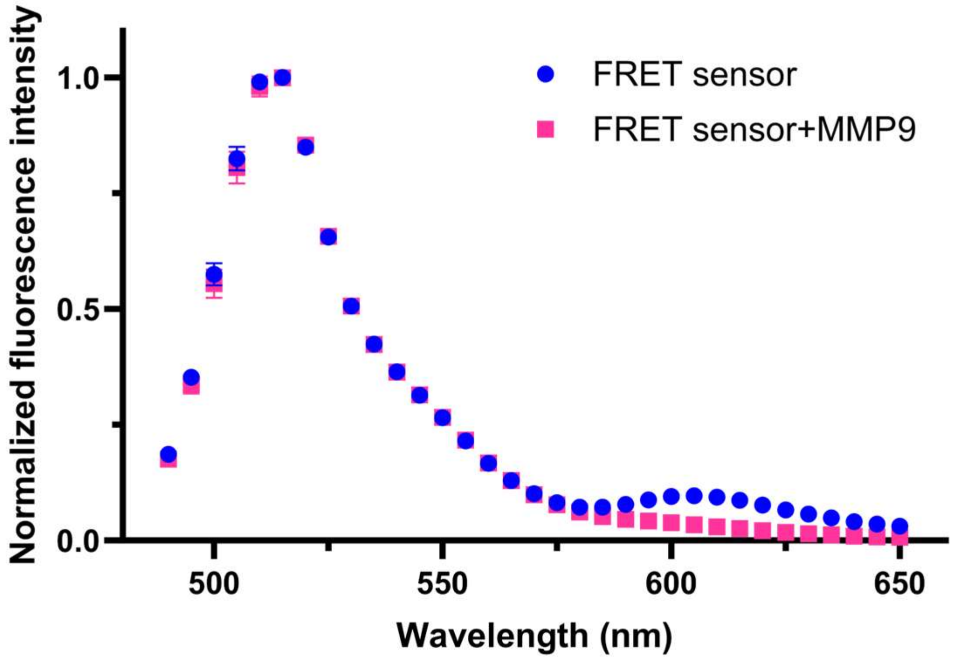

b

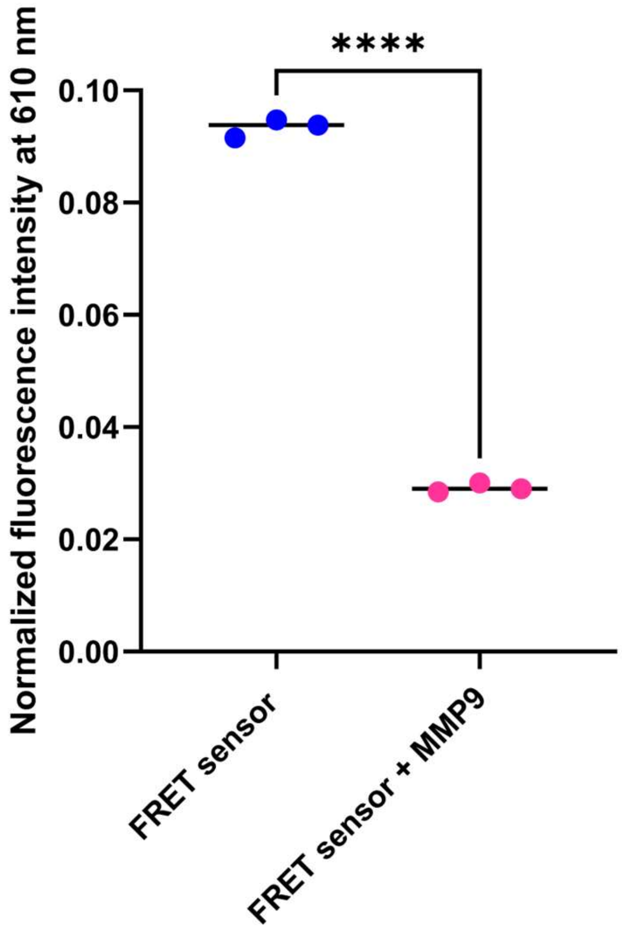

Supplementary Fig. 5: **a**. Normalized mean fluorescence intensity (± S.D.) of the emission spectra of the FRET sensor with and without 1 h treatment with MMP9. SFGFP was excited at 462 nm (bandwidth: 16 nm) and the emission captured between 490 and 650 nm. **b**. Normalized mean fluorescence intensity of the emission spectra at 610 nm (peak emission of mRFP) collected from FRET sensor with or without 1 h MMP9 treatment after excitation at 462 nm. The fluorescence intensity was significantly lower in the MMP9-treated group compared to the untreated control (n=3 for both groups; ****p<0.0001, unpaired two-tailed t-test).

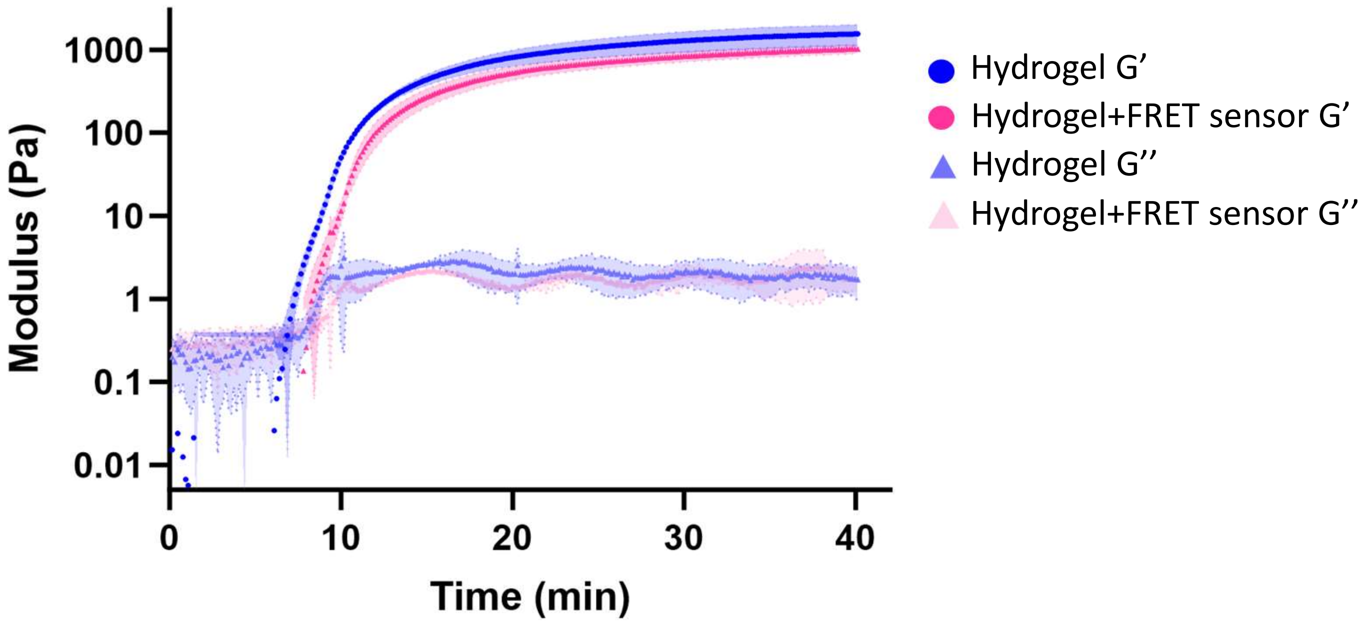

Supplementary Fig. 6: Time sweep rheological measurements of the gelation reaction to form hydrogels with and without the FRET sensor. Data are presented as means (dots) with the shaded area showing standard deviation. Mean plateau modulus (G’) was no different in hydrogels containing the FRET sensor compared to standard hydrogels without the FRET sensor (p = 0.12, unpaired two-tailed t-test, n=3).

a

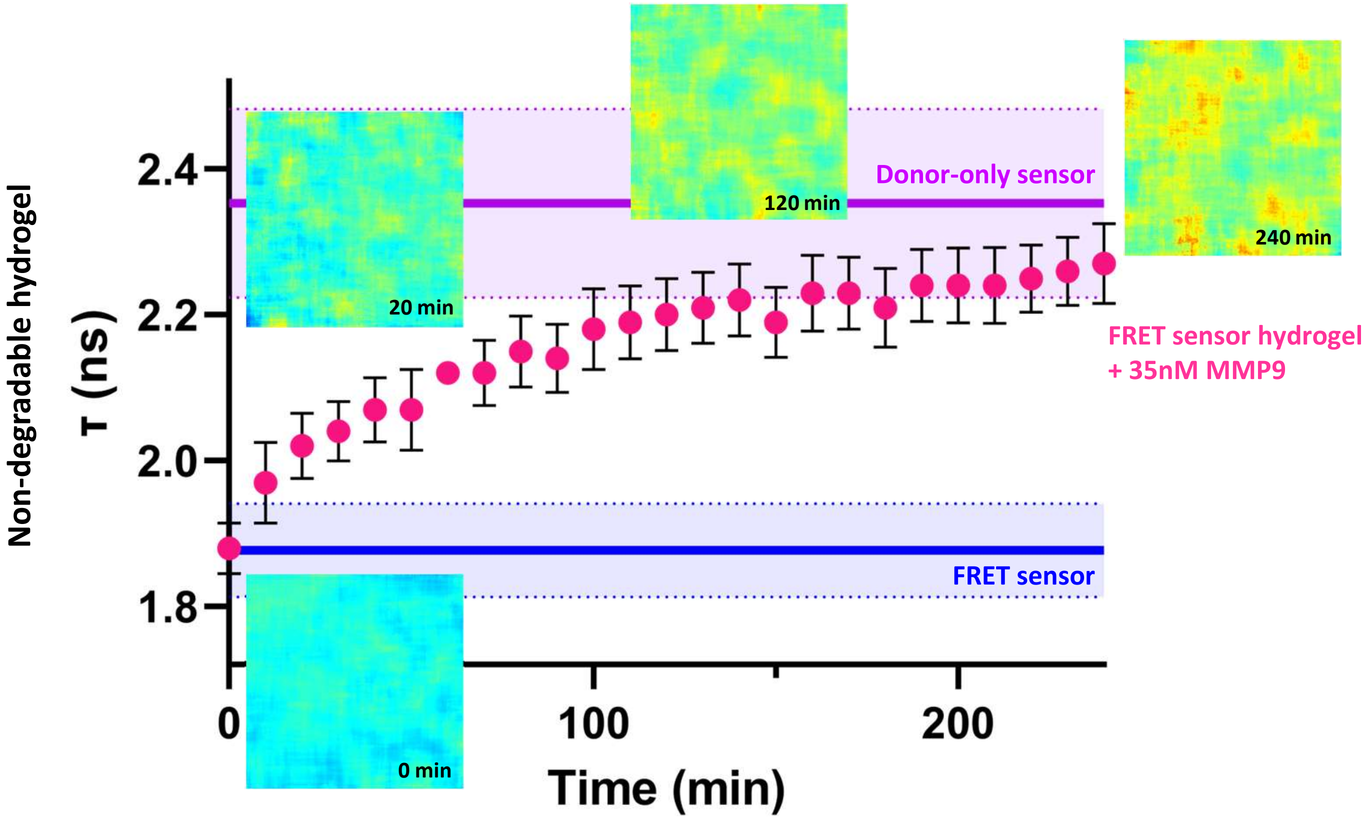

b

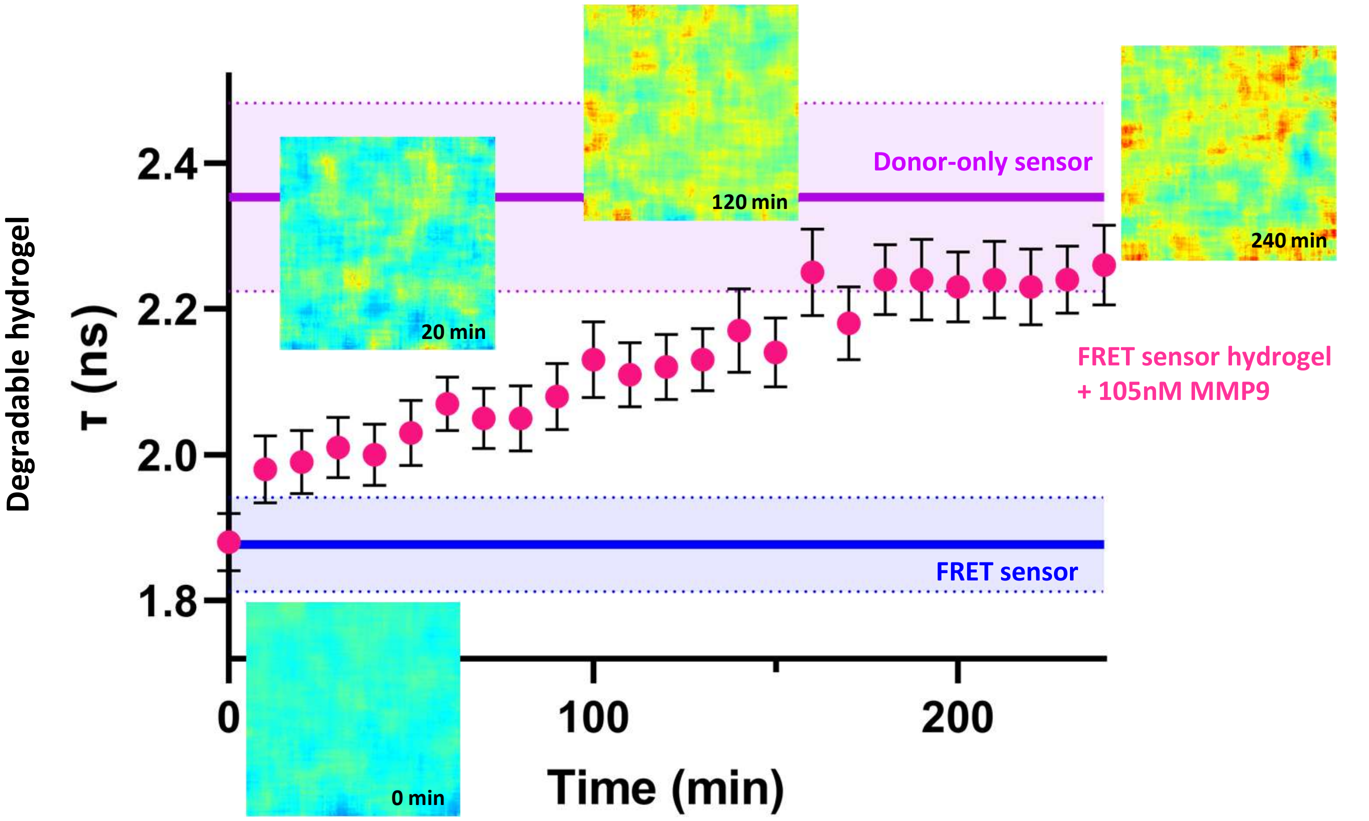

Supplementary Fig. 7 a. Mean τ (± S.D.) measured over 240 min for non-degradable hydrogels containing the FRET sensor treated with 35nM MMP9, and **b**. degradable hydrogels treated with 105nM MMP9, reproduced from Fig. 3c and overlaid with example heat maps. Mean τ (±S.D., shaded area) of the FRET sensor and donor-only sensor are shown for comparison (Donor-only sensor, FRET sensor n=3; FRET sensor hydrogel+MMP9 n=4)

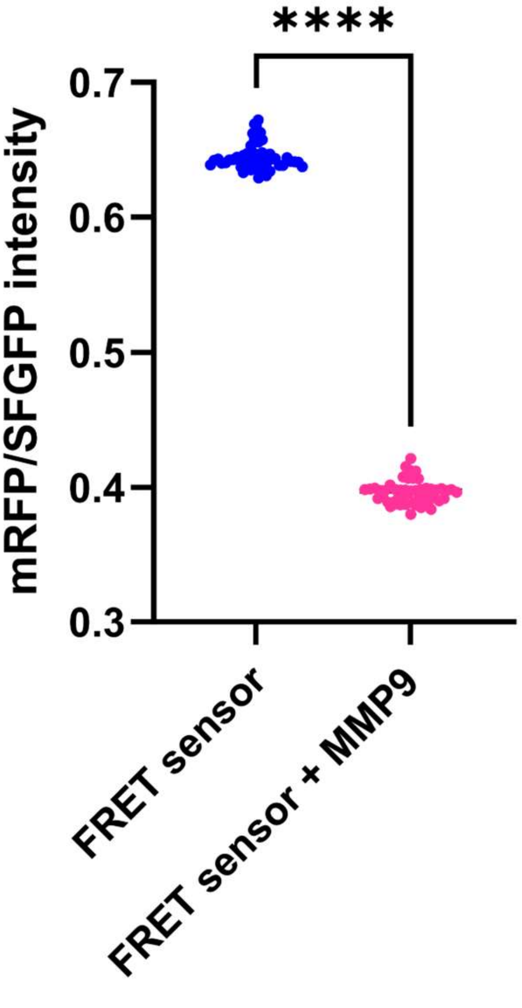

Supplementary Fig. 8: Fluorescence intensity ratios of mRFP:SFGFP measured on FRET sensor-containing hydrogels either without (FRET sensor) or with 4 h treatment with 35 nM MMP9 (FRET sensor + MMP9). The fluorescence intensity ratio was significantly lower in MMP9-treated hydrogels compared to the untreated control (****p<0.0001, unpaired two-tailed t-test).

a

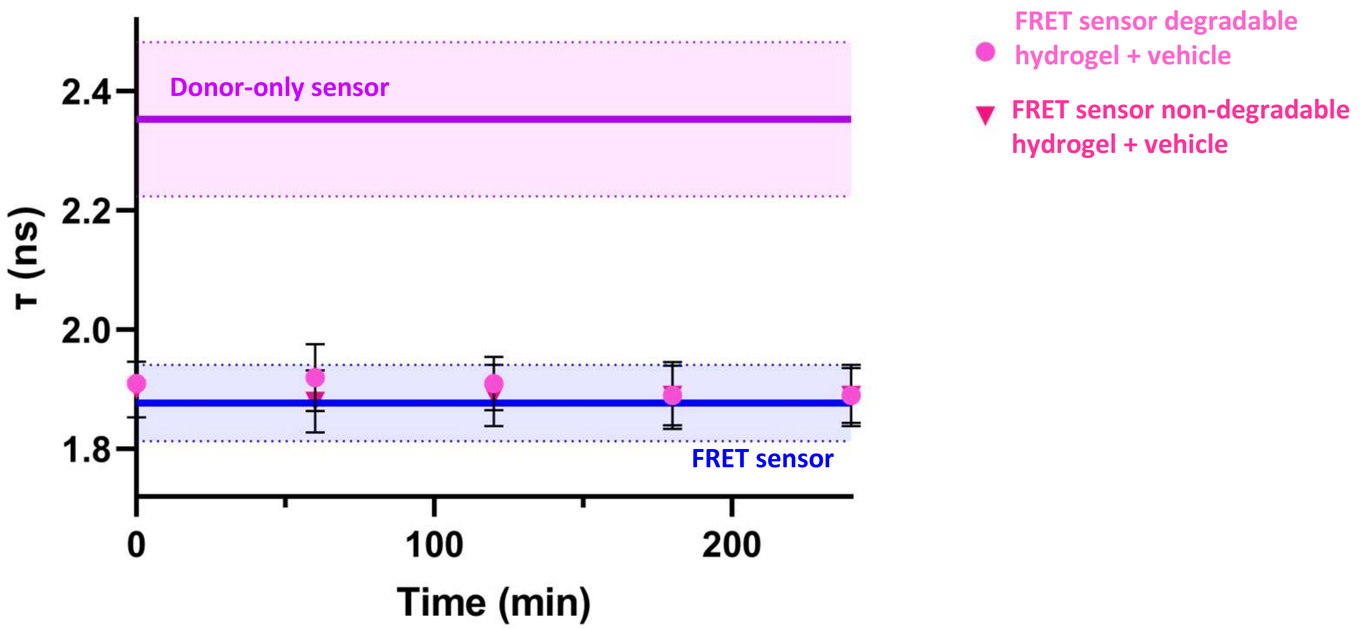

b

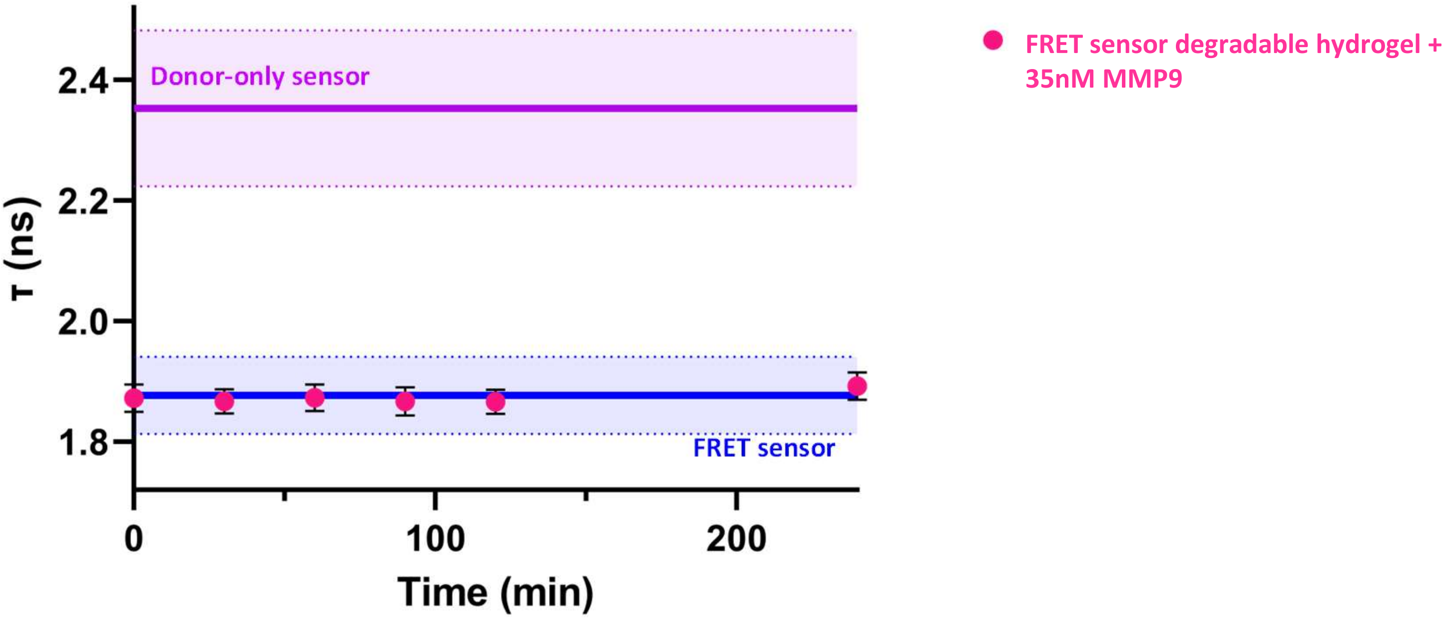

Supplementary Fig. 9: **a**. Mean τ (± S.D.) measured over 240 min for non-degradable hydrogels containing the FRET sensor and degradable hydrogels containing the FRET sensor, both treated with the vehicle control (no MMP9). Mean τ (±S.D., shaded area) of the FRET sensor and donor-only sensor are shown for comparison (Donor-only sensor, FRET sensor n=3; FRET sensor degradable and non-degradable hydrogels n=2). **b**. Mean τ (± S.D.) measured over 240 min for degradable hydrogel containing the FRET sensor treated with 35nM MMP9. Mean τ (±S.D., shaded area) of the FRET sensor and donor-only sensor are shown for comparison (Donor-only sensor, FRET sensor n=3; FRET sensor degradable and non-degradable hydrogels n=2).

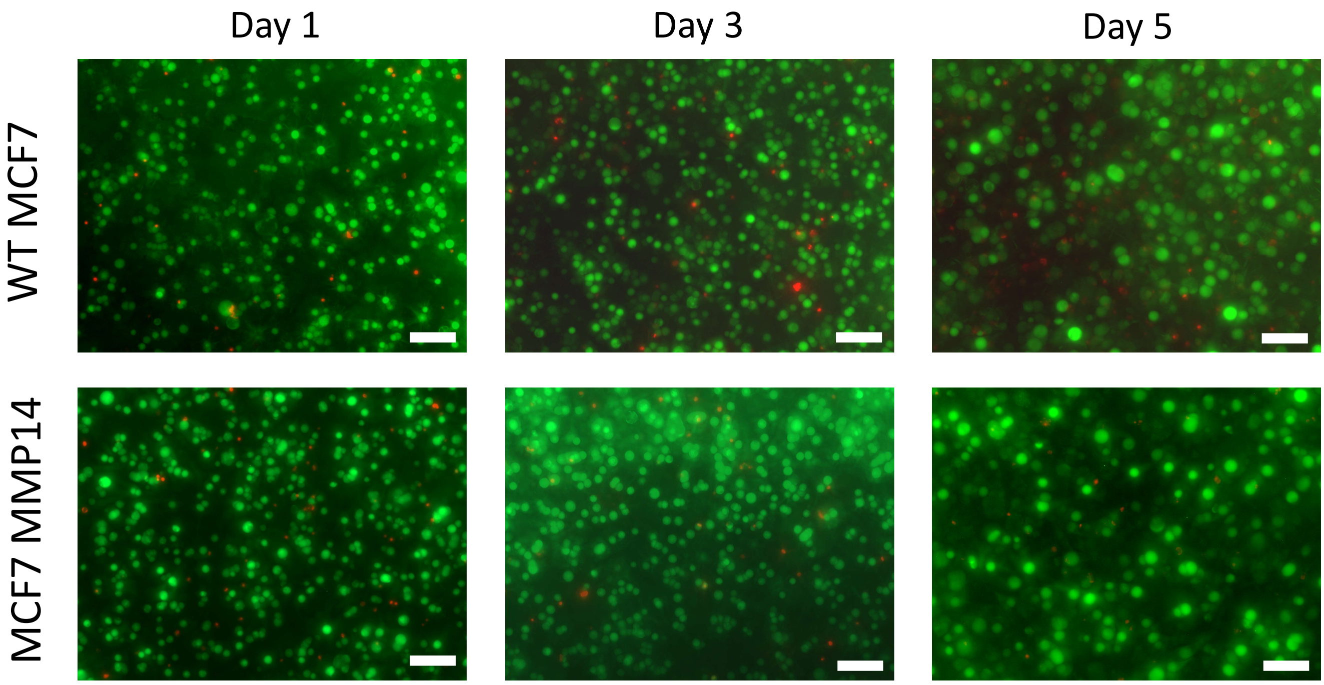

Supplementary Fig. 10: Representative fluorescence images of calcein AM (green, live) and ethidium homodimer (red, dead) staining of WT MCF7 and MCF7 MMP14 cells within non-degradable PEG hydrogels after 1, 3 and 5 days in culture. Scale bar = 100 µm.

atgcatcatcaccatcaccatggtaagcctatccctaaccctctcctcggtctcgattctacggaaaacctgtattttcagggaattga
tcccttcaccatggcctcctccgaggacgtcatcaaggagttcatgcgcttcaaggtgcgcatggagggctccgtgaacggccacga
gttcgagatcgagggcgagggcgagggccgcccctacgagggcacccagaccgccaagctgaaggtgaccaagggcggcccccct
gcccttcgcctgggacatcctgtcccctcagttccagtacggctccaaggcctacgtgaagcaccccgccgacatccccgactacttg
aagctgtccttccccgagggcttcaagtgggagcgcgtgatgaacttcgaggacggcggcgtggtgaccgtgacccaggactcctc
cctgcaggacggcgagttcatctacaaggtgaagctgcgcggcaccaacttcccctccgacggccccgtaatgcagaagaagacc
atgggctgggaggcctccaccgagcggatgtaccccgaggacggcgccctgaagggcgagatcaagatgaggctgaagctgaag
gacggcggccactacgacgccgaggtcaagaccacctacatggccaagaagcccgtgcagctgcccggcgcctacaagaccgac
atcaagctggacatcacctcccacaacgaggactacaccatcgtggaacagtacgagcgcgccgagggccgccactccaccggcg
ccccgtcatcaggtggaggtagctctggaggtggctcctctggtggaggtagtggtccacagggtatctggggtcagagtggaggc
ggtagctcaggcggtggtagtagtggtggaggtagcatggtgagcaaaggtgaagaactgtttacgggcgttgtgccgattttggtc
gaactggatggtgatgtaaatgggcataaattctcggtccgcggtgaaggtgagggggatgcgacttatgggaagctgacccttaa
gtttatctcgaccaccggcaaactgccggtgccctggccgacgctggtgacgacgttaacttacggtgtgcagatgttcgcgcgctat
cccgatcatatgaagcagcacgatttttttaaatcggccatgccggaaggttatgtgcaggaacgcaccatctttttcaaggatgacg
gcaattacaaaacgcgtgcagaagttaaatttgaaggcgacactctggtgaaccgtatcgagcttaaaggaattgatttcaaagaa
gatggcaatatcttgggccacaagcttgaatacaactacaattcacataacgtatatattaccgcggataaacagaaaaacggaat
taaggcgaatttcaagattcgtcataacatcgaagatggtggcgtccagctggctgaccactaccaacagaacaccccaatcggcg
acggcccggtgctgctgccggataatcactatttgtctacacagagcaagctgagtaaagatccgaatgaaaaacgggatcatatg
gtgttgcttgagtttgtcaccgcggctggcatcggaggtggtggaagtcgctgttaa

Supplementary Table 1: Encoding sequence for FRET protein.

atgcatcatcaccatcaccatggtaagcctatccctaaccctctcctcggtctcgattctacggaaaacctgtattttcagggaattgatc
ccttcacctcaggtggaggtagctctggaggtggctcctctggtggaggtagtggtccacagggtatctggggtcagagtggaggcgg
tagctcaggcggtggtagtagtggtggaggtagcatggtgagcaaaggtgaagaactgtttacgggcgttgtgccgattttggtcgaac
tggatggtgatgtaaatgggcataaattctcggtccgcggtgaaggtgagggggatgcgacttatgggaagctgacccttaagtttatc
tcgaccaccggcaaactgccggtgccctggccgacgctggtgacgacgttaacttacggtgtgcagatgttcgcgcgctatcccgatca
tatgaagcagcacgattttttaaatcggccatgccggaaggttatgtgcaggaacgcaccatctttttcaaggatgacggcaattaca
aaacgcgtgcagaagttaaatttgaaggcgacactctggtgaaccgtatcgagcttaaaggaattgatttcaaagaagatggcaatat
cttgggccacaagcttgaatacaactacaattcacataacgtatatattaccgcggataaacagaaaaacggaattaaggcgaatttc
aagattcgtcataacatcgaagatggtggcgtccagctggtcgctgaccactaccaacagaacacccccaatcggcgacggcccggtg
ctgctgccggataatcactatttgtctacacagagcaagctgagtaaagatccgaatgaaaaacgggatcatatggtgttgcttgagtt
tgtcaccgcggctggcatcggaggtggtggaagtcgctgttaa

Supplementary Table 2: Encoding sequence for donor-only protein.

a

Number of FRET constructs tethered to PEG	MW of PEG-FRET construct conjugate (kDa)	Theoretical elution volume (mL)
0	20	
1	78	14.05
2	136	12.77
3	194	11.95
4	252	11.35
5	310	10.87
6	368	10.47
7	326	10.13
8	484	9.84

b

Number of donor-only constructs tethered to PEG	MW of PEG-donor only construct conjugate (kDa)	Theoretical elution volume (mL)
0	20	
1	53	14.94
2	86	13.83
3	119	13.08
4	152	12.51
5	185	12.06
6	218	11.68
7	251	11.35
8	284	11.07

Supplementary Table 3: **a.** MW and theoretical elution volume of PEG molecules conjugated with up to 8 FRET constructs. **b**. MW and theoretical elution volume of PEG molecules conjugated with up to 8 donor-only constructs.

a

Hydrogel type (non-degradable/ adhesive)	Hydrogel polymer conc. (%)	Hydrogel volume (µL)	Molar conc. of PEG-peptide conjugates (µM)	Molar conc. of non-functional peptide (µM)	Molar conc. of RGD-containing peptide (µM)	Molar conc. of PEG-4VS (µM)	Molar conc. of VS groups (PEG-4VS) (µM)	Molar conc. of FRET sensor (µM)
FRET-sensor containing	2.5	30	707	2262	566	706	2824	1
No FRET sensor	2.5	30	707	2262	566	706	2828	0

b

Hydrogel type (degradable /adhesive)	Hydrogel polymer conc. (%)	Hydrogel volume (µL)	Molar conc. of PEG-peptide conjugates (µM)	Molar conc. of MMP-degradable peptide (µM)	Molar conc. of non-functional peptide (µM)	Molar conc. of RGD-containing peptide (µM)	Molar conc. of PEG-4VS (µM)	Molar conc. of VS groups (PEG-4VS) (µM)	Molar conc. of FRET sensor (µM)
FRET-sensor containing	2.5	30	673	1211.4	942.2	538.4	672	2688	1
No FRET sensor	2.5	30	673	1211.4	942.2	538.4	673	2692	0

Supplementary Table 4: Description of the components of hydrogels formed either with the FRET sensor or without. **a**. Calculation for a non-degradable hydrogel formed with the RGD sequence-containing peptide (RGDSGDKGDQGIAGFERC) and the non-functional peptide (KDWERC). **b**. Calculation for a degradable hydrogel formed with the MMP-degradable peptide (GRDSGKGPQGIWGQERC), the RGD sequence-containing peptide (RGDSGDKGDQGIAGFERC) and the non-functional peptide (KDWERC).
